# Pictilisib and nutrient stress synergize to induce methuosis via PI(4,5)P_2_-dependent macropinocytic dysregulation in cancer cells

**DOI:** 10.1038/s41419-026-08994-6

**Published:** 2026-06-16

**Authors:** Xuefei Wang, Xiaolin Sang, Yuemei Wen, Xingming Liao, Jiao Liu, Tiezheng Zheng, Tianming Xing, Min Wang, Dong An, Xining Zhang, Xiuling Li, Xinmiao Liang, Pixu Liu, Hailing Cheng

**Affiliations:** 1 Cancer Institute, The Second Hospital of Dalian Medical University, Dalian, Liaoning China; 2 Zhejiang Key Laboratory of Intelligent Cancer Biomarker Discovery and Translation, First Affiliated Hospital, Wenzhou Medical University, Wenzhou, Zhejiang China; 3 Institute of Cancer Stem Cell, Dalian Medical University, Dalian, Liaoning China; 4 Key Laboratory of Phytochemistry and Natural Medicines, Dalian Institute of Chemical Physics, Chinese Academy of Sciences, Dalian, Liaoning China; 5 Ganjiang Chinese Medicine Innovation Center, Nanchang, Jiangxi China; 6 Dalian Lingshui Bay Laboratory, Dalian, Liaoning China

**Keywords:** Cell death, Cancer

## Abstract

Aberrant phosphatidylinositol 3-kinase (PI3K) activation drives many cancers, but PI3K inhibitors like Pictilisib often induce cytostasis rather than cytotoxicity, limiting their therapeutic potential. Here we demonstrate that PI3K inhibition combined with nutrient stress triggers methuosis, a non-apoptotic form of programmed cell death characterized by dysregulated macropinosomes. This response occurs selectively in PI3K-aberrant cancer cells that maintain macropinocytic uptake despite PI3K inhibition. Methuosis-associated vacuoles originate from macropinosomes that retain endosomal markers but fail to undergo lysosomal fusion. Active macropinocytic uptake is essential for methuosis, as demonstrated by suppression with EIPA and Bafilomycin A1, whereas the AKT inhibitor MK2206 has no effect, establishing that direct PI3K inhibition, rather than AKT signaling, is required. Mechanistically, PI3K blockade prevents conversion of phosphatidylinositol (4,5)-bisphosphate (PI(4,5)P_2_) to phosphatidylinositol (3,4,5)-trisphosphate (PI(3,4,5)P_3_) causing PI(4,5)P_2_ to accumulate on internalizing macropinosomal membranes. This aberrant PI(4,5)P_2_ enrichment impairs ion channel function across multiple channel families, disrupting intracellular osmotic balance. Ion dysregulation triggers aquaporin-1-mediated water influx, driving catastrophic vacuolar expansion and cell death. Although Pictilisib activates pro-survival autophagy, this fails to prevent methuosis-mediated cytotoxicity. In xenograft models, dietary restriction synergizes with Pictilisib to suppress tumor growth, correlating with pronounced intratumoral vacuolization. These findings reveal that combining PI3K inhibition with nutrient restriction converts cytostatic responses into methuosis-driven cytotoxicity via PI(4,5)P_2_-dependent macropinocytic dysregulation, providing a rational pharmacologic-dietary strategy to enhance PI3K-targeted cancer efficacy.

## Introduction

The phosphatidylinositol 3-kinase (PI3K) pathway is a critical regulator of essential cellular processes, including proliferation, survival, and metabolism [1]. The pathway operates through the phosphorylation of phosphatidylinositol (4,5)-bisphosphate (PI(4,5)P_2_) to generate phosphatidylinositol (3,4,5)-trisphosphate (PI(3,4,5)P_3_), which recruits and activates downstream effectors including AKT via PDK1, thereby promoting cell growth and survival [2]. Dysregulation of PI3K signaling is among the most frequently occurring oncogenic alterations in human cancers, typically driven by activating mutations in *PIK3CA* (e.g. E545K or H1047R) or loss-of-function mutations in the tumor suppressor *PTEN* [3–5]. Given this central role in oncogenesis, PI3K has emerged as an attractive target for cancer therapy. However, clinical development of PI3K inhibitors, including the pan-class I agent Pictilisib, has been hampered by limited efficacy as monotherapies or combination regimens. These agents typically induce cytostasis (cell cycle arrest) rather than cytotoxicity [6–9], resulting in disease stabilization rather than tumor regression. Overcoming this limitation, converting PI3K inhibitors from cytostatic to cytotoxic agents, remains a major challenge in cancer therapy.

Recent advances have highlighted non-apoptotic cell death mechanisms as alternative therapeutic strategies, particularly for apoptosis-resistant cancers [10–13]. Methuosis, a term derived from the Greek “*methuo”* for intoxication, represents one such mechanism. It is characterized by excessive cytoplasmic vacuolization arising from dysregulated macropinocytosis and distinct from classical apoptotic pathways [14–16]. Macropinocytosis is an actin-driven endocytic process involving membrane ruffling and invagination that enables cells to engulf extracellular fluid and nutrients [17–19]. Under nutrient-stressed conditions, characteristic of poorly vascularized tumor microenvironments with high metabolic demands [20, 21], cancer cells frequently upregulate macropinocytosis to sustain cell survival and proliferation [22–24]. However, when uncontrolled, excessive macropinocytic activity leads to the pathological accumulation of large, non-lysosomal vacuoles [16]. This accumulation causes progressive cellular swelling, disruption of critical organelles, and ultimately membrane rupture and cell death. We hypothesized that nutrient restriction could amplify the effects of methuosis-inducing agents by increasing cancer cells’ dependence on macropinocytosis, thereby potentiating their vulnerability to dysregulated vesicular trafficking.

Here, we demonstrate that combining PI3K inhibition with nutrient restriction induces extensive vacuolization and methuosis-like cell death specifically in cancer cells with aberrant PI3K pathway activation. Using both in vitro and in vivo models, we show that PI3K inhibitors administered under nutrient-deprived conditions trigger dysregulated macropinocytosis and robust cytotoxicity. Notably, this approach converts Pictilisib, a pan-class I PI3K inhibitor with limited single-agent efficacy, into a potent cytotoxic agent. Our findings establish a mechanistically rational framework for combining pharmacological PI3K inhibition with dietary restriction, offering a promising strategy to enhance the clinical efficacy of PI3K-targeted therapies.

## Materials and methods

### Cell culture and reagents

All cell lines used in this study were obtained from the American Type Culture Collection and authenticated using short tandem repeat profiling at study initiation. Mycoplasma contamination was routinely monitored to ensure cell fidelity. The following cell lines were cultured in specific media. SNU119, OVCAR4, CaSki, MDA-MB-468, HCC70, HCC1954 and PC-3 cells were maintained in RPMI-1640. OVSAHO cells were cultured in DMEM. ME180 cells were grown in McCoy’s 5 A (modified) medium. All media were supplemented with 10% fetal bovine serum and 100 U/ml Penicillin/Streptomycin (Gibco, USA). Cells were kept in a humidified incubator at 37 °C with 5% CO_2_. All media were purchased from Invitrogen. The following reagents were utilized: Pictilisib and MK2206 (BioChemPartner, China); Bafilomycin A1, EIPA, Z-VAD-FMK, MRT68921, m-3M3FBS, NPPB, ML133, SET2, ML141, FRAX597, BEZ235, NSC23766, and Cisplatin (MedChemExpress, USA); N-Acetyl-L-cysteine (NAC) and puromycin (Sigma-Aldrich, USA). Stable overexpression of *PIK3CA*^E545K^ in OVCAR4 cells was achieved using the retroviral vector pBabe-puro-HA-PIK3CA-E545K (#12525, Addgene, USA).

### PI(4,5)P_2_ measurement

To evaluate cellular PI(4,5)P_2_ localization, cells were transfected with a GFP-tagged PH domain of PLCδ-expressing plasmid (pLV3-CMV-EGFP-PLCδ-PH-Puro, P50541, MiaoLing Biology, China) using Lipofectamine 3000 (Invitrogen) for 48 h. Following transfection, cells were treated with or without Pictilisib for 24 h in serum-free medium, fixed with 4% formaldehyde, and imaged using a DM6B Thunder fluorescence microscope (Leica Microsystems, Germany) to visualize PI(4,5)P_2_ localization.

### siRNA transfection

siRNA oligonucleotides were transfected into cells using Lipofectamine 3000 (Invitrogen) according to the manufacturer’s instructions. siRNA reagents were purchased from GenePharma (China). The sequences used were:

siAQP1-sense: 5’-GCCAUCCUCUCAGGCAUCACCUCCU-3’

siAQP1-anti-sense: 5’-AGGAGGUGAUGCCUGAGAGGAUGGC-3’

siMKK4-sense: 5’-GCCUUACGAAGGAUGAAUCCATT-3’

siMKK4-anti-sense: 5’-UGGAUUCAUCCUUCGUAAGGCTT-3’

### Quantitative reverse transcription PCR (qRT-PCR)

Total RNA was isolated using the TRIzol Reagent (Invitrogen) according to the manufacturer’s instructions and reverse-transcribed into complementary DNA (cDNA) using the PrimeScript RT reagent Kit (Takara Bio Inc, Japan). qRT-PCR was performed using SYBR Green PCR Master Mix (Applied Biosystems, USA) on a StepOnePlus Real-Time PCR system (Applied Biosystems, USA). Primer sequences were synthesized by Sangon Biotech and are as follows:

MKK4-sense: 5′-ACAGGAGTTCAAAACCCACAC-3′

MKK4-antisense: 5′-TCCCAGTGTTGTTCAGGGGA-3′

*ACTB* was used as an endogenous control.

### Western blot analysis

Cell lysates were prepared using ice-cold RIPA buffer supplemented with protease and phosphatase inhibitors (Roche, Switzerland). Proteins were separated by SDS-PAGE, transferred to nitrocellulose membranes, and probed with the following primary antibodies: phospho-AKT Ser473 (CST#4060, Cell Signaling Technology, USA), phospho-AKT Thr308 (CST#2965), AKT (CST#9272), phospho-S6RP Ser235/Ser236 (CST#4858), Phospho-PAK1 (Ser199/204)/PAK2 (Ser192/197) (CST#2605), SQSTM1/p62 (PTM#6434, PTM Biolabs Inc, China), LC3B (PTM#6384), phospho-SEK1/MKK4 Ser257/Thr261 (CST#9156), Aquaporin 1 (ab168387, Abcam, UK), cleaved PARP (CST#9541), and Vinculin (V9131, Sigma). Fluorescence-conjugated secondary antibodies were purchased from Li-COR (Lincoln, USA). Blots were imaged using the Odyssey Infrared Imaging System (Li-COR).

### Transmission electron microscopy analysis

OVSAHO cells, with or without 5 μM Pictilisib treatment under serum starvation, were fixed in 2.5% glutaraldehyde (pH 7.4) and subjected to transmission electron microscopy analysis. Following fixation, cells were washed three times with 0.1 M phosphate buffer (pH 7.2) and post-fixed in 1% osmium tetroxide at 4 °C for 2 h. Samples were dehydrated in graded ethanol, embedded in Epon-Araldite resin, and polymerized. Semi-thin sections were prepared for orientation, followed by ultrathin sections for detailed analysis. Sections were stained with 3% uranyl acetate and 2.7% lead citrate. Images were acquired using a JEM-1400 transmission electron microscope (JEOL Ltd., Japan).

### Live cell imaging

The uptake of the fluid-phase fluorescent tracer Lucifer Yellow (LY; L0144, Sigma-Aldrich) for labeling intracellular vacuoles was performed as previously described [15]. Briefly, cells were treated with or without Pictilisib for 24 h, followed by incubation with LY (1 mg/mL) for 2 h at 37 °C. Acidic intracellular compartments were labeled with Lyso-Tracker Red (L12492, Thermo Fisher Scientific) at 50 nM for 30 min. Phase-contrast and fluorescence images were acquired using a confocal microscope (TCS SP8, Leica Microsystems). Cell morphology was analyzed using a phase-contrast microscope (DMi1, Leica Microsystems). Four representative fields per treatment condition were analyzed for imaging analysis.

### Dextran uptake assays

Cells were seeded onto glass coverslips placed in 24-well plates. Twenty-four hours after seeding, the cells were treated with or without Pictilisib in serum-free medium for 24 h. To label macropinosomes, a high-molecular-weight tetramethylrhodamine (TMR)-dextran (D1818, Thermo Fisher Scientific) uptake assay was conducted. TMR-dextran was added to the serum-free medium at a final concentration of 1 mg/mL and incubated with the cells for 30 min at 37 °C. Following incubation, the cells were washed five times with cold PBS and immediately fixed with 3.7% formaldehyde. The fixed cells were then stained with DAPI in PBS for 10 min, followed by three additional washes with PBS. Fluorescence images were captured using a DM6B Thunder fluorescence microscope (Leica Microsystems). Image analysis and quantification were performed as previously described [25].

### Immunofluorescence microscopy

Cells were fixed, permeabilized, and blocked prior to incubation with primary antibodies overnight at 4 °C. Fluorescence-conjugated secondary antibodies (Thermo Fisher Scientific) and DAPI solution (Sigma-Aldrich) were used for visualization. The primary antibodies included EEA1 (sc-137130, Santa Cruz, USA), Rab7 (CST#95746), PI(4,5)P_2_ (ab11039, Abcam). Fluorescence images were acquired using a DM6B Thunder fluorescence microscope (Leica Microsystems).

### Measurement of autophagy flux

Stable overexpression of mRFP1-EGFP-LC3B in cancer cells was achieved using the lentiviral vector pCDH-CMV-mRFP1-EGFP-LC3B-EF1-Puro (P4838, MiaoLing Biology). Following transduction, cells were selected with puromycin to establish stable cell lines. Cells were then treated with the indicated drugs, fixed with 4% formaldehyde, and imaged using a DM6B Thunder fluorescence microscope (Leica Microsystems). For quantification of autophagy flux, more than 30 cells were analyzed per treatment group, and the number of yellow and red puncta per cell was counted.

### Cell viability assays

Cells were treated with the indicated drugs, with media replaced every other day. At the endpoint, cells were fixed and stained with 0.5% crystal violet solution. Bound dye was solubilized with 50% acetic acid, and optical density was measured at 590 nm using an xMark Microplate Absorbance Spectrophotometer (Bio-Rad Laboratories, USA).

### Flow cytometry analysis

For cell death analysis, cells were stained with Propidium Iodide following the manufacturer’s instructions (Sigma). Briefly, cells were harvested, washed with PBS, and incubated with Propidium Iodide solution to assess membrane integrity. Stained cells were analyzed using a BD FACS Canto II flow cytometer (BD Biosciences, USA). For intracellular Reactive oxygen species (ROS) measurement, cells were incubated with 5 μM H2DCFDA (#D399, Invitrogen, USA) for 1 h at 37 °C. After incubation, cells were washed with PBS, and fluorescence was analyzed using the BD FACS Canto II flow cytometer to quantify ROS levels.

### RNA sequencing and proteomics analysis

For RNA sequencing analysis, OVSAHO and SNU119 cells were treated with or without Pictilisib (5 µM) for 24 h in serum-free medium. Total RNA was isolated using TRIzol Reagent (Invitrogen) for RNA sequencing analysis, which was performed by Novogene. Sequencing libraries were generated using the NEBNext® UltraTM RNA Library Prep Kit for Illumina® (NEB) according to the manufacturer’s protocol. Gene Set Enrichment Analysis (GSEA) was conducted to identify Gene Ontology Molecular Function (GO:MF) pathways associated with Pictilisib treatment in OVSAHO and SNU119 cells. The analysis was performed using the JAVA program (http://software.broadinstitute.org/gsea/index.jsp) with the Molecular Signatures Database (MSigDB). A total of 1000 random sample permutations were performed, with significance thresholds set at *p* < 0.01 and false discovery rate (FDR) < 0.05.

For proteomics analysis, OVSAHO and SNU119 cells, treated with or without 5 µM Pictilisib for 24 h in serum-free medium, were lysed in a buffer containing 8 M urea, 1% protease inhibitor cocktail, and 1 mM phenylmethylsulfonyl fluoride (PMSF). The extracted proteins were digested with trypsin and analyzed using liquid chromatography-tandem mass spectrometry (LC-MS/MS). Peptides were separated on a C18 column using an EASY-nLC 1200 system coupled to an Orbitrap Exploris™ 480 mass spectrometer (Thermo Fisher Scientific). The mobile phases consisted of 0.1% formic acid (A) and 80% acetonitrile with 0.1% formic acid (B). The gradient elution program was as follows: 3–15% B in 0.5 min, 15–50% B in 15 min, 50–90% B in 1.5 min, and 90% B for 5 min. The flow rate was maintained at 800 nL/min throughout the analysis. Data-independent acquisition (DIA) was performed with a precursor mass range of 350–800 m/z, using 59 windows of 6.7 m/z and a normalized collision energy. Data were collected in profile mode with positive polarity. Peptide match was turned off, and isotope exclusion was enabled to improve data quality. Raw data were processed using Spectronaut 19 (Biognosys AG, Switzerland) against the UniProt-Human database.

For integrated proteomics and transcriptomic data analysis, peptides identified by mass spectrometry or transcripts with a log2 Fold Change > 0.5 and a *p* < 0.05 were considered differentially expressed proteins (DEPs) or differentially expressed genes (DEGs), respectively. Gene Ontology Cellular Component (GO:CC) pathway enrichment analysis was conducted using the ClusterProfiler 4.0 R package [26].

### In vivo mouse xenograft study

Eight-week-old female BALB/c nude mice (Beijing Vital River Laboratory Animal Technology) were subcutaneously inoculated with 4 × 10^6^ SNU119 cells mixed with Matrigel (BD Biosciences). Once tumors reached approximately 70–80 mm^3^, the mice were randomized into four groups: ad libitum feeding (AL), dietary-restricted (DR), Pictilisib under ad libitum conditions (Pictilisib + AL), and Pictilisib treatment combined with dietary restriction (Pictilisib + DR). The AL group was fed an irradiated purified rodent chow diet (AIN-93M, Jiangsu Xietong Pharmaceutical Bio-Engineering) with a physiological fuel value of 3.601 kcal/g, consisting of 14.1% protein, 10% fat, and 72.7% carbohydrates. The DR group received a daily meal providing 60% of the caloric intake of the AL group. To prevent nutrient deficiencies, the restricted diet was enriched with minerals and vitamins (XT19008, Jiangsu Xietong Pharmaceutical Bio-Engineering) while maintaining a similar consistency to the ad libitum diet. Pictilisib was dissolved in 0.5% methylcellulose/0.2% Tween 80 and administered via oral gavage at a dose of 120 mg/kg once daily for the indicated treatment period. Tumor volumes were measured every other day using calipers and calculated as: tumor volume = (length × width^2^)/2.

### Histological analysis

Tumors were fixed in 10% neutral-buffered formalin overnight and subsequently embedded in paraffin. Paraffin-embedded blocks were sectioned and stained with hematoxylin and eosin (H&E) for histological analysis. Stained sections were examined under a light microscope to assess histopathological features.

### Statistical analysis

Statistical analysis was conducted using unpaired Student’s *t* test, one-way ANOVA with Tukey’s multiple comparisons test, or two-way ANOVA with Bonferroni’s Tukey’s multiple comparisons test (GraphPad Prism). Data represent the Mean ± S.D. from three independent experiments or Mean ± S.E.M. for mouse xenograft study. *p* < 0.05 were considered statistically significant.

## Results

### PI3K inhibition triggers lethal vacuolization in serum-starved cancer cells with sustained macropinocytosis

**Fig. 1:**
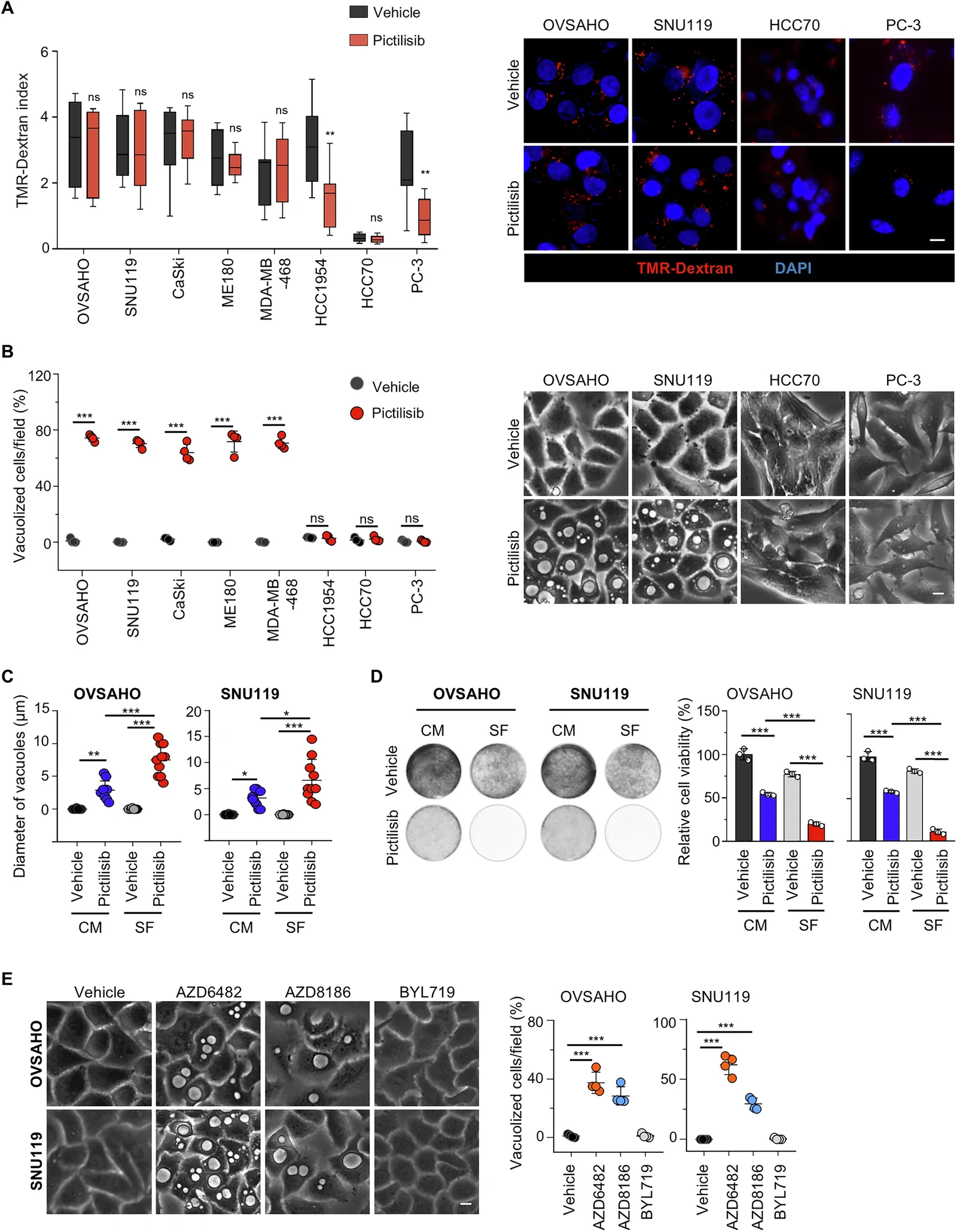
PI3K inhibition induces cytoplasmic vacuolization in PI3K-driven cancers with sustained macropinocytic activity. **A** Quantification of TMR-dextran index in multiple cancer cell lines with or without Pictilisib for 24 h (*n* = 8). Representative fluorescence images are shown. Scale bar, 10 µm. **B** Quantification of Pictilisib-induced cytoplasmic vacuolization after 24 h (*n* = 4). Representative phase-contrast images are shown. Scale bar, 10 µm. **C** Quantification of vacuole diameter in OVSAHO and SNU119 cells treated with Pictilisib for 24 h in complete medium (CM) or serum-free medium (SF) (*n* = 10). **D** Clonogenic survival of OVSAHO and SNU119 cells cultured in complete or serum-free culture conditions, treated with or without Pictilisib for 7 days (*n* = 3). **E** Phase-contrast images of cells treated with AZD6482 (50 µM), AZD8186 (50 µM), and BYL719 (50 µM) for 24 h (*n* = 4). Scale bar, 10 µm. Pictilisib, 5 µM. **A**–**E** Data are shown as mean ± SD and representative of three independent experiments. **A**, **B** ns not significant, **p < 0.01, ***p < 0.001 (Student’s *t*-test). **C**–**E** *p < 0.05, **p < 0.01, ***p < 0.001 (One-way ANOVA with Tukey’s multiple comparisons test).

We investigated whether Class I PI3Ks are required for macropinocytosis during nutrient stress by measuring tetramethylrhodamine (TMR)-dextran uptake in serum-starved cancer cell lines harboring diverse PI3K pathway alterations. Among five cell lines with constitutive PI3K signaling (CaSki and ME180 with *PIK3CA*^E545K^, MDA-MB-468 with *PTEN* deletion, and OVSAHO and SNU119 with elevated phosphorylated AKT [27]), TMR-dextran uptake persisted despite treatment with the pan-Class I PI3K inhibitor Pictilisib (Fig. 1A and Supplementary Fig. 1A), demonstrating that these cells maintain PI3K-independent macropinocytosis under nutrient stress. Strikingly, Pictilisib induced prominent cytoplasmic vacuolization exclusively in these same five cell lines (Fig. 1B and Supplementary Fig. 1B), accompanied by substantial cell death and loss of viability (Supplementary Fig. 1C, D). In contrast, three additional *PTEN*-altered or *PIK3CA*-mutant lines (HCC70 with *PTEN* deletion; HCC1954 with *PIK3CA*^H1047R^; and PC-3 with *PTEN* deletion) exhibited either no detectable dextran uptake or significantly reduced macropinocytosis upon PI3K inhibition (Fig. 1A and Supplementary Fig. 1A) and importantly, none developed vacuolization (Fig. 1B and Supplementary Fig. 1B). This striking correlation suggests that sustained macropinocytic uptake despite PI3K blockade is a prerequisite for the vacuolization phenotype.

To determine whether nutrient stress is obligatory for this response, we treated OVSAHO and SNU119 cells with Pictilisib in complete medium versus serum-free conditions. Pictilisib alone in complete medium induced only modest vacuolization with moderate reduction in cell viability (Fig. 1C, D, Supplementary Fig. 2), whereas the combination of serum starvation and Pictilisib produced extensive vacuolization with markedly enlarged vacuoles and near-complete suppression of cell growth (Fig. 1C, D, Supplementary Fig. 2). Thus, nutrient stress is necessary for robust Pictilisib-induced cell death.

To identify which PI3K isoforms drive this response, we employed isoform-selective inhibitors in serum-starved OVSAHO, SNU119, and MDA-MB-468 cells. PI3Kβ-selective inhibition by AZD6482, and to a lesser extent by AZD8186, provoked pronounced vacuolization (Fig. 1E, Supplementary Fig. 3), whereas the PI3Kα-selective inhibitor BYL719 did not. Treatment with BEZ235, a dual pan-PI3K/mTOR inhibitor, similarly induced vacuolization under serum starvation (Supplementary Fig. 4), though the contribution of mTOR inhibition cannot be separated from that of PI3K inhibition in these experiments. These findings indicate that PI3K inhibition triggers lethal cytoplasmic vacuolization in nutrient-stressed cells with PI3K-independent macropinocytosis, and we pursued further mechanistic investigation using the pan-Class I PI3K inhibitor Pictilisib.

**Fig. 2:**
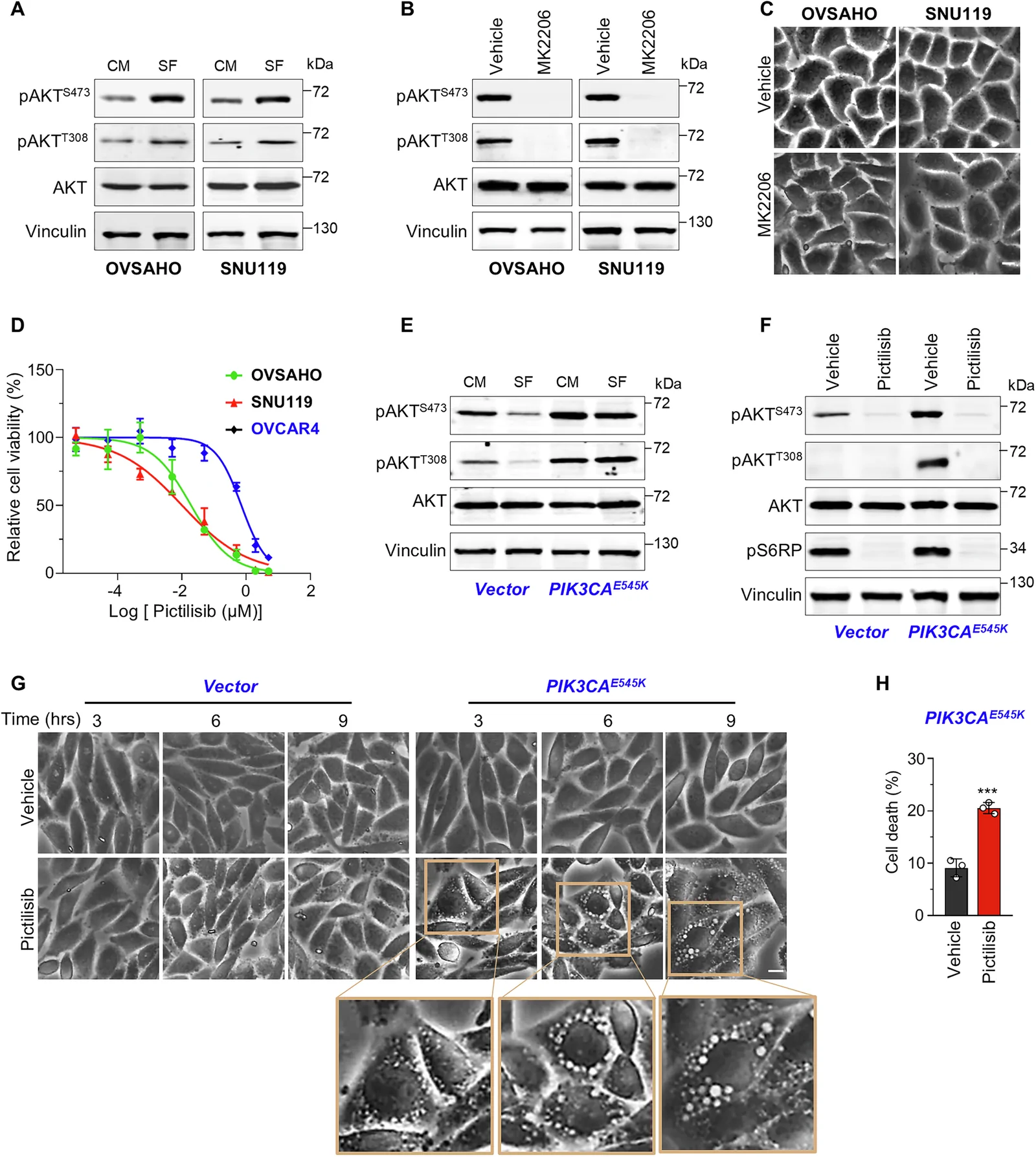
Pictilisib induces AKT-independent vacuolization in PI3K-activated cancers. **A** Western blot analysis of indicated proteins in OVSAHO and SNU119 cells cultured with or without serum for 24 h. **B** Western blot analysis of indicated proteins in OVSAHO and SNU119 cells treated with MK2206 (2.5 µM) for 24 h. **C** Phase-contrast images corresponding to (**B**) (*n* = 4). Scale bar, 10 μm. **D** Cell viability after 7 days of treatment with or without Pictilisib in complete medium (*n* = 3). **E** Western blot analysis of indicated proteins in *vector* or *PIK3CA*^E545K^-expressing OVCAR4 cells cultured with or without serum for 24 h. **F** Western blot analysis of indicated proteins in *vector* or *PIK3CA*^E545K^-expressing OVCAR4 cells treated with or without Pictilisib (5 µM) for 24 h. **G** Phase-contrast images of *vector* or *PIK3CA*^E545K^-expressing OVCAR4 cells treated with Pictilisib (5 µM) for the indicated time points. Scale bar, 10 µm. **H** Cell death in *PIK3CA* ^E545K^-expressing OVCAR4 cells treated with or without Pictilisib (5 µM) for 48 h. Data are shown as mean ± SD (*n* = 3) and are representative of three independent experiments. *** *p* < 0.001 (Student’s *t*-test). **A,** **B,** **E,** **F** Vinculin was used as a loading control. **B,** **C,** **F-H** All experiments were conducted under serum-free conditions.

To investigate whether inhibition of AKT, a key downstream effector in the PI3K signaling [1], contributes to the observed vacuolization, we first confirmed that AKT phosphorylation was effectively suppressed by Pictilisib (pan-Class I inhibitor), AZD6482 (p110 β inhibitor), AZD8186 (p110β inhibitor) or BYL719 (p110 α inhibitor) in OVSAHO and SNU119 cells under serum-deprived conditions (Supplementary Figs. 2B and 3A, B). Notably, serum starvation alone increased AKT activation (Fig. 2A). Treatment with the allosteric AKT inhibitor MK2206 robustly inhibited AKT activity (Fig. 2B) but did not induce cytoplasmic vacuolization (Fig. 2C). Thus, the vacuolization elicited by PI3K inhibition occurs through an AKT-independent mechanism.

To evaluate the contribution of aberrant PI3K activation to Pictilisib-induced vacuolization, we engineered OVCAR4 cells, which lack constitutive PI3K activation and are relatively insensitive to Pictilisib compared with OVSAHO or SNU119 cells (Fig. 2D), to stably express the constitutively active *PIK3CA*^E545K^ mutation. This conferred persistent PI3K pathway signaling irrespective of serum availability, while AKT activation remained suppressible by Pictilisib (Fig. 2E, F). In this setting, Pictilisib provoked marked cytoplasmic vacuolization and significantly increased cell death (Fig. 2G, H). Collectively, these findings demonstrate that under nutrient stress, PI3K inhibition induces cytoplasmic vacuolization and cell death in a subset of PI3K-driven cancer cells via an AKT-independent mechanism, with sensitivity linked to maintenance of macropinocytic uptake.

### Vacuoles induced by Pictilisib exhibit characteristics of macropinosomes

**Fig. 3:**
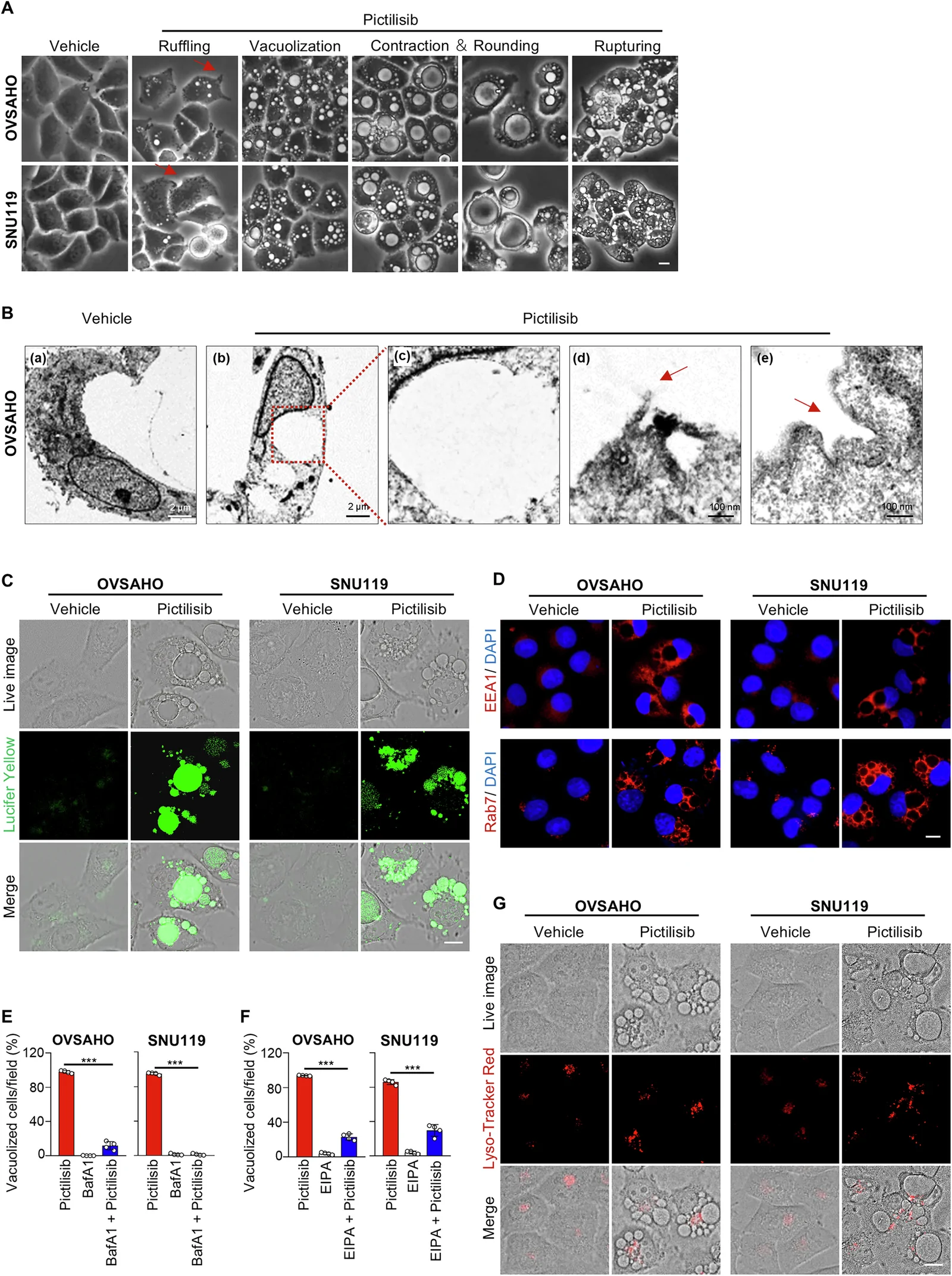
Vacuoles induced by Pictilisib exhibit characteristics of macropinosomes. **A** Representative phase-contrast of OVSAHO and SNU119 cells showing membrane ruffling (red arrows), vacuolization, contraction, rounding, and rupture after Pictilisib treatment. Scale bar, 10 µm. **B** Transmission electron microscopy of OVSAHO cells treated with or without Pictilisib for 24 h. a Vehicle-treated cells with preserved cytoplasmic compartments. b Pictilisib-treated cells with extensive vacuolization. c Pictilisib-induced vacuoles are optically clear and bound by a single membrane. d Red arrow indicates plasma membrane-bound vacuoles and irregular protrusions relative to vehicle. e Red arrow indicates cup-shaped plasma membrane invaginations. **C** Cells treated with or without Pictilisib for 24 h were incubated with Lucifer Yellow (LY, 1 mg/ml) for 2 h at 37 °C. Scale bar, 10 µm. **D** Immunofluorescence staining of EEA1 and Rab7 in cells treated with or without Pictilisib for 24 h. Scale bar, 10 µm. Quantification of vacuolized OVSAHO and SNU119 cells pretreated with Bafilomycin A1 (2 nM, **E**) or EIPA (25 μM, **F**), followed by co-treatment with or without Pictilisib for 24 h (*n* = 4). Data are shown as mean ± SD and representative of three independent experiments. ***p < 0.001 (One-way ANOVA with Tukey’s multiple comparisons test). **G** Cells treated with or without Pictilisib for 24 h were incubated with Lyso-Tracker Red (50 nM) for 30 min at 37 °C. Scale bar, 10 µm. **A**–**G** All experiments were conducted under serum-free conditions. Pictilisib, 5 µM.

Given that macropinocytic uptake persisted in subsets of PI3K-driven cancer cell lines despite PI3K inhibition (Fig. 1A), we hypothesized that the observed cytoplasmic vacuolization reflects dysregulated macropinosomes and undertook a detailed characterization of their formation and properties. We first monitored the temporal dynamics of vacuole development under serum starvation. Vacuoles emerged within hours of Pictilisib exposure, progressively enlarged, and culminated in plasma-membrane rupture and cell death (Fig. 3A). Transmission electron microscopy (TEM) after 24 h of treatment revealed large, single-membrane vacuoles devoid of cytosolic contents or organelles (Fig. 3Ba–c), distinguishing them from double-membrane autophagosomes that sequester cytoplasmic material. TEM also captured irregular plasma-membrane protrusions and invaginations (Fig. 3Bd, e), consistent with active macropinocytosis.

To confirm the macropinocytic origin, we employed Lucifer Yellow (LY), a fluid-phase tracer of macropinosomes [15, 28]. Fluorescence microscopy showed that most vacuoles were LY-positive (Fig. 3C). Colocalization with the early endosomal marker EEA1 and late endosomal marker Rab7 (Fig. 3D) indicated endocytic maturation. Further mechanistic validation came from Bafilomycin A1, a vacuolar-type H^+^-ATPase inhibitor [29, 30], which abolished Pictilisib-induced vacuolization (Fig. 3E, Supplementary Fig. 5A). Similarly, 5-(N-ethyl-N-isopropyl) amiloride (EIPA), a Na^+^/H^+^ exchanger inhibitor that disrupts macropinosome biogenesis [31], significantly attenuated vacuole formation (Fig. 3F, Supplementary Fig. 5B). Notably, Lyso-Tracker Red staining revealed no colocalization with lysosomes (Fig. 3G), indicating that these macropinosomes fail to undergo lysosomal fusion.

### Autophagy is induced by PI3K inhibition but is insufficient to counter methuotic cytotoxicity

**Fig. 4:**
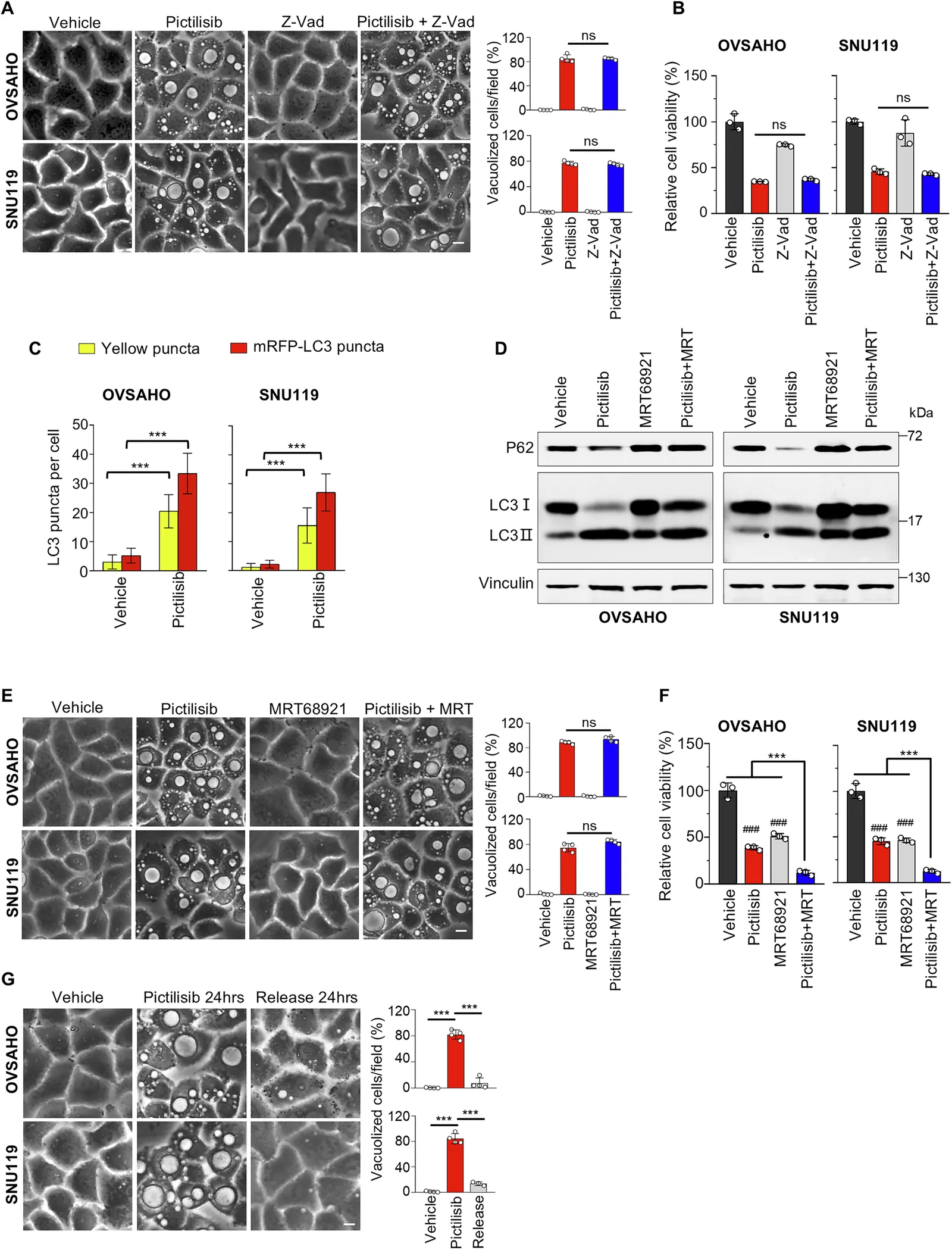
Pictilisib induces caspase-independent methuosis and promotes autophagic flux. **A** Phase-contrast images of OVSAHO and SNU119 cells pretreated with or without Z-VAD-FMK (Z-Vad, 20 μM), followed by co-treatment with or without Pictilisib for 24 h (*n* = 4). Scale bar, 10 µm. **B** Cell viability of cells treated as in (**A**) for 5 days (*n* = 3). **C** Cells stably expressing mRFP-GFP-LC3 were treated with or without Pictilisib for 24 h. Quantification of yellow puncta (both mRFP and GFP; autophagosomes) and red puncta (mRFP only; autolysosomes) per cell is shown (*n* = 30). **D** Western blot analysis of indicated proteins in cells pretreated with MRT68921 (1.5 μM), followed by co-treatment with or without Pictilisib for 24 h. Vinculin was used as a loading control. **E** Phase-contrast images corresponding to (**D**) after 24 h (*n* = 4). Scale bar, 10 µm. **F** Cell viability of cells treated as in (**D**) for 48 h (*n* = 3). **G** Phase-contrast images of cells treated with Pictilisib for 24 h and then switched to serum-free medium for an additional 24 h after drug removal (*n* = 4). Scale bar, 10 µm. **A**–**G** All experiments were conducted under serum-free conditions. Pictilisib, 5 µM. Data are shown as mean ± SD and representative of three independent experiments. **C** ***p < 0.001 (Two-way ANOVA with Tukey’s multiple comparisons test). **A**, **B**, **E**–**G** ^##**#**^ *p* < 0.001 (*vs* vehicle); ***p < 0.001. ns not significant (One-way ANOVA with Tukey’s multiple comparisons test).

To further characterize the death modality, we assessed apoptotic involvement. The pan-caspase inhibitor Z-VAD-FMK did not prevent vacuolization or the associated loss of viability induced by Pictilisib under nutrient stress (Fig. 4A, B, Supplementary Fig. 6A), confirming methuosis as a non-apoptotic form of cell death. Because suppression of the PI3K/AKT/mTOR axis is a well-established trigger of autophagy [32], we next evaluated autophagic flux. In cells expressing the mRFP-GFP-LC3 tandem reporter, Pictilisib elicited a pronounced increase in both yellow (autophagosomes) and red (autolysosomes) puncta (Fig. 4C, Supplementary Fig. 6B). Immunoblotting corroborated autophagy induction, showing enhanced LC3-I to LC3-II conversion and depletion of the autophagy adaptor p62/SQSTM1 [33] (Fig. 4D). To determine whether autophagy modulates vacuolization-associated cytotoxicity, we co-treated cells with MRT68921, a ULK1/2 inhibitor that blocks autophagy initiation [34]. MRT68921 had minimal impact on vacuole accumulation (Fig. 4D, E) but significantly reduced cell viability in OVSAHO and SNU119 cells (^###^; Fig. 4F). Moreover, Pictilisib combined with MRT68921 produced an additive reduction in viability compared with either agent alone (^***^; Fig. 4F). These results indicate that vacuolization is autophagy-independent, whereas autophagy affords partial cytoprotection against methuosis stress, and its blockade sensitizes cells to Pictilisib-mediated cytotoxicity. Notably, vacuolization was reversible, as vacuoles dissipated upon Pictilisib withdrawal after 24 h (Fig. 4G), indicating that sustained PI3K inhibition is required to commit cells irreversibly to this death modality.

Collectively, these data demonstrate that Pictilisib induces vacuolization in nutrient-stressed PI3K-driven cancer cells through dysregulated macropinocytosis, generating enlarged, non-lysosomal macropinosomes distinct from autophagic compartments. Although PI3K inhibition activates autophagy as a cytoprotective response, this survival mechanism is insufficient to prevent methuotic cell death.

### Aberrant PI(4,5)P_2_ accumulation on macropinosomal membranes drives Pictilisib-induced vacuolization via ion channel dysregulation

**Fig. 5:**
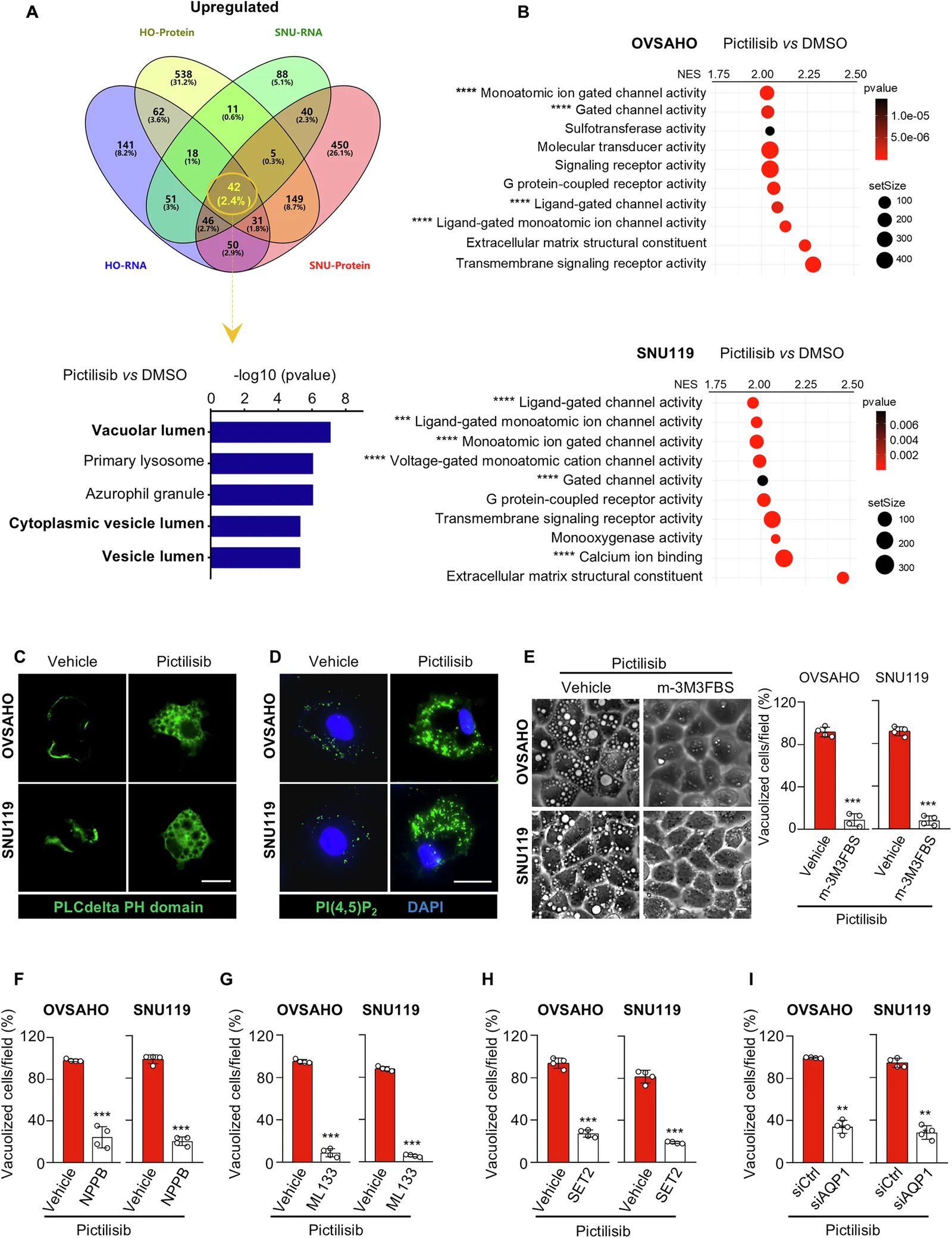
Aberrant enrichment of PI(4,5)P_2_ on macropinosomal membranes contributes to Pictilisib-induced methuosis. **A** Venn diagram showing upregulated proteins (DEPs, HO-Protein and SNU-Protein) and genes (DEGs, HO-RNA and SNU-RNA) in OVSAHO and SNU119 cells treated with Pictilisib (5 µM, 24 h). Gene Ontology Cellular Component (GO:CC) enrichment was performed on the 42 entities upregulated at both mRNA and protein levels in both cell lines. **B** RNA-seq analysis identifying the top ten Gene Ontology Molecular Function (GO:MF) pathways associated with Pictilisib treatment in OVSAHO and SNU119 cells. **C** Cells transfected with GFP-tagged PLCδ PH domain plasmid for 48 h, followed by treatment with Pictilisib for 24 h. Representative GFP images are shown. Scale bar, 20 µm. **D** Immunofluorescence staining of PI(4,5)P_2_ in cells treated with or without Pictilisib for 24 h. Scale bar, 20 µm. **E** Cells pretreated with or without m-3M3FBS (12.5 μM) were subsequently treated with or without Pictilisib for 24 h. Scale bar, 10 µm. Quantification of vacuolized OVSAHO and SNU119 cells pretreated with NPPB (80 μM, **F**), ML133 (25 μM, **G**), or SET2 (10 μM, **H**), followed by co-treatment with or without Pictilisib for 24 h. **I** Quantification of vacuolized OVSAHO and SNU119 cells transfected with siCtrl or siAQP1 for 48 h, followed by co-treatment with or without Pictilisib for 24 h. **A**–**I** All experiments were conducted under serum-free conditions. Pictilisib, 5 μM. **E**–**I** Data are shown as mean ± SD (*n* = 4) and representative of three independent experiments. ***p < 0.001 (Student’s *t* test).

To investigate the molecular mechanisms underlying Pictilisib-induced vacuoles, we performed integrated proteomic and transcriptomic analyses in Pictilisib-treated OVSAHO and SNU119 cells. Venn diagram analysis identified 42 genes consistently upregulated at both the mRNA and protein levels in both cell lines (Fig. 5A and Supplementary Data 1). Pathway enrichment highlighted vacuolar compartments, including the vacuolar lumen and cytoplasmic vesicle lumen (Fig. 5A and Supplementary Data 2), consistent with the observed methuosis-like vacuolization phenotype. Notably, Gene Set Enrichment Analysis (GSEA) revealed enrichment of ion channel-related gene sets, including “ligand-gated monoatomic ion channel activity” and “gated channel activity” (Fig. 5B), implicating ion channel dysregulation as a key mechanistic feature of methuosis and directing our attention to the interplay between lipid signaling, membrane dynamics, and ion transport on macropinosomal membranes.

Phosphatidylinositol (4,5)-bisphosphate (PI(4,5)P_2_) is a direct PI3K substrate and master regulator of ion channel activity and membrane dynamics [35–37]. Under Pictilisib treatment, PI(4,5)P_2_ cannot be converted to PI(3,4,5)P_3_ and therefore accumulates at the plasma membrane. This accumulated PI(4,5)P_2_ is continuously internalized into macropinosomes via bulk membrane uptake, resulting in progressive enrichment on vacuolar membranes. To establish the temporal relationship between PI(4,5)P_2_ dynamics and vacuole formation, we performed time-lapse phase-contrast imaging combined with immunofluorescence in Pictilisib-treated cells. PI(4,5)P_2_ accumulation on macropinosomal membranes preceded morphologically detectable giant vacuole formation (Supplementary Fig. 7), indicating a causative role. This finding was independently confirmed using a GFP-tagged PLCδ PH domain biosensor [38] and direct PI(4,5)P_2_ immunostaining (Fig. 5C, D, Supplementary Fig. 8A), both showing aberrant PI(4,5)P_2_ retention specifically on vacuolar membranes. To confirm that PI(4,5)P_2_ accumulation is functionally required for methuosis, rather than a passive consequence, we depleted PI(4,5)P_2_ by activating PLCδ with m-3M3FBS, which hydrolyzes PI(4,5)P_2_ into diacylglycerol (DAG) and inositol trisphosphate (IP_3_) [39]. This intervention significantly suppressed vacuolization in OVSAHO and SNU119 cells (Fig. 5E), and in CaSki, ME180, MDA-MB-468, and OVCAR4-*PIK3CA*^E545K^ cell lines (Supplementary Fig. 8B). Together, these data establish that PI(4,5)P_2_ accumulation on macropinosomal membranes is both temporally upstream of and mechanistically required for Pictilisib-induced methuosis.

PI(4,5)P_2_ electrostatically regulates the gating and conductance of multiple ion-transporting protein families, including inward-rectifier K⁺ channels (Kir), transient receptor potential (TRP) channels, and chloride channels [36, 37]. Its aberrant enrichment on macropinosomal membranes is therefore expected to broadly impair coordinated ion flux, disrupting the osmotic equilibrium required for normal vesicle maturation and recycling [16]. Consistent with this model, pharmacological inhibition of structurally and functionally distinct channel families, using the chloride channel blocker NPPB, the Kir2.X inhibitor ML133, or the TRPV2 antagonist SET2, each markedly suppressed Pictilisib-induced vacuolization (Fig. 5F–H, Supplementary Fig. 9A–C). The convergent rescue effect across these mechanistically diverse inhibitors argues that PI(4,5)P_2_ accumulation acts through broad, non-specific disruption of ion homeostasis across multiple channel types rather than through engagement of any single channel.

Dysregulated ion retention within macropinosomes generates osmotic pressure that drives water influx. We therefore examined Aquaporin-1 (AQP1), a transmembrane water channel that facilitates rapid osmotic water movement [40, 41]. AQP1 knockdown profoundly suppressed Pictilisib-induced vacuolization (Fig. 5I, Supplementary Fig. 9D, E), providing direct functional evidence that AQP1-mediated water flux is the terminal effector of vacuolar expansion. Together, these findings delineate a mechanistic cascade in which PI(4,5)P_2_ accumulation on macropinosomal membranes broadly disrupts ion channel function, generates intravacuolar osmotic pressure, and drives AQP1-mediated water influx to produce the methuotic vacuolar phenotype.

To identify the signaling pathway sustaining macropinocytic uptake, a critical initial step for methuosis, under PI3K inhibition, we systematically examined canonical regulators by measuring fluorescent dextran uptake in Pictilisib-treated OVSAHO and SNU119 cells following targeted inhibition of RAC1, CDC42, and PAK [16, 42]. Inhibition of RAC1 or CDC42 produced only minimal suppression of dextran uptake (Supplementary Fig. 10A), indicating these upstream GTPases are largely dispensable under PI3K inhibition. In striking contrast, PAK inhibition substantially reduced dextran uptake (Supplementary Fig. 10A), identifying PAK as the primary driver of macropinosome biogenesis in this context. Notably, consistent with this functional requirement, Pictilisib-mediated PI3K inhibition resulted in increased PAK phosphorylation (Supplementary Fig. 10B), supporting sustained PAK activation during PI3K blockade. These results establish that PI3K-inhibited cells sustain PAK-dependent macropinocytotic uptake independently of the canonical Rac1/CDC42 pathway.

**Fig. 6:**
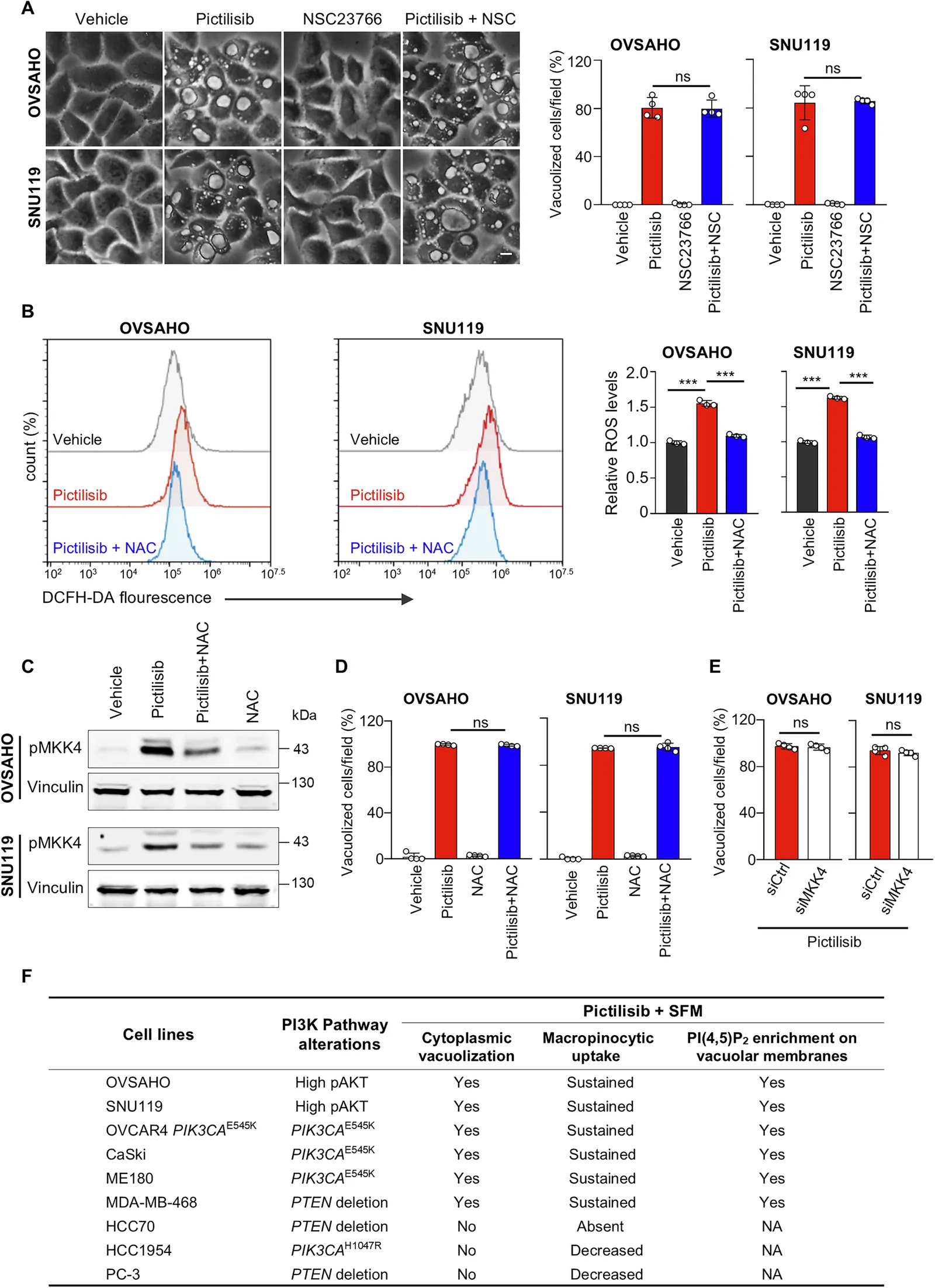
Pictilisib-induced methuosis is independent of the RAS-RAC and ROS-MKK4 pathway. **A** Phase-contrast images of cancer cells pretreated with the Rac1 inhibitor NSC23766 (10 μM), followed by co-treatment with Pictilisib for 24 h (*n* = 4). Scale bar, 10 µm. **B** ROS levels in cells pretreated with NAC (4 mM) and co-treated with or without Pictilisib for 48 h (*n* = 3). **C** Western blot analysis of phosphorylated MKK4 in cells treated as in (**B**) for 48 h. Vinculin was used as a loading control. **D** Quantification of cytoplasmic vacuolization in cells treated as in (**B**) for 24 h (*n* = 4). **E** Quantification of cytoplasmic vacuolization in cells transfected with siCtrl or siMKK4 and treated with Pictilisib for 24 h (*n* = 4). **F** Summary table of PI3K pathway alterations and the status of cytoplasmic vacuolization, macropinocytic uptake, and PI(4,5)P_2_ enrichment on vacuolar membranes across the indicated cell lines after Pictilisib treatment in serum-free medium (SFM). NA, not applicable. **A**–**F** All experiments were conducted under serum-free conditions. Pictilisib, 5 μM. Data are shown as mean ± SD and representative of three independent experiments. **A**, **B**, **D**, **E** ns not significant, ***p < 0.001 (One-way ANOVA with Tukey’s multiple comparisons test).

Methuosis has been attributed to Ras-Rac1 and reactive oxygen species (ROS)-driven MKK4 activation in other cellular contexts [15, 43–45], raising the question of whether these alternative pathways contribute to Pictilisib-induced vacuolization. Rac1 inhibition with NSC23766 [46] failed to suppress vacuole formation (Fig. 6A), excluding a Rac1-dependent mechanism. Similarly, although Pictilisib elevated both ROS levels and MKK4 phosphorylation (Fig. 6B, C), neither antioxidant treatment with N-acetyl-L-cysteine (NAC) nor MKK4 knockdown prevented vacuolization (Fig. 6D, E, Supplementary Fig. 11), ruling out ROS and MKK4 as contributory factors. These findings establish that Pictilisib-induced PI(4,5)P_2_ accumulation on macropinosomal membranes drives methuosis by disrupting ion channel function, compromising osmotic homeostasis, and promoting AQP1-mediated water influx in PI3K-dependent cancer cells (Fig. 6F).

### Pictilisib synergizes with dietary restriction to induce methuosis in vivo

**Fig. 7:**
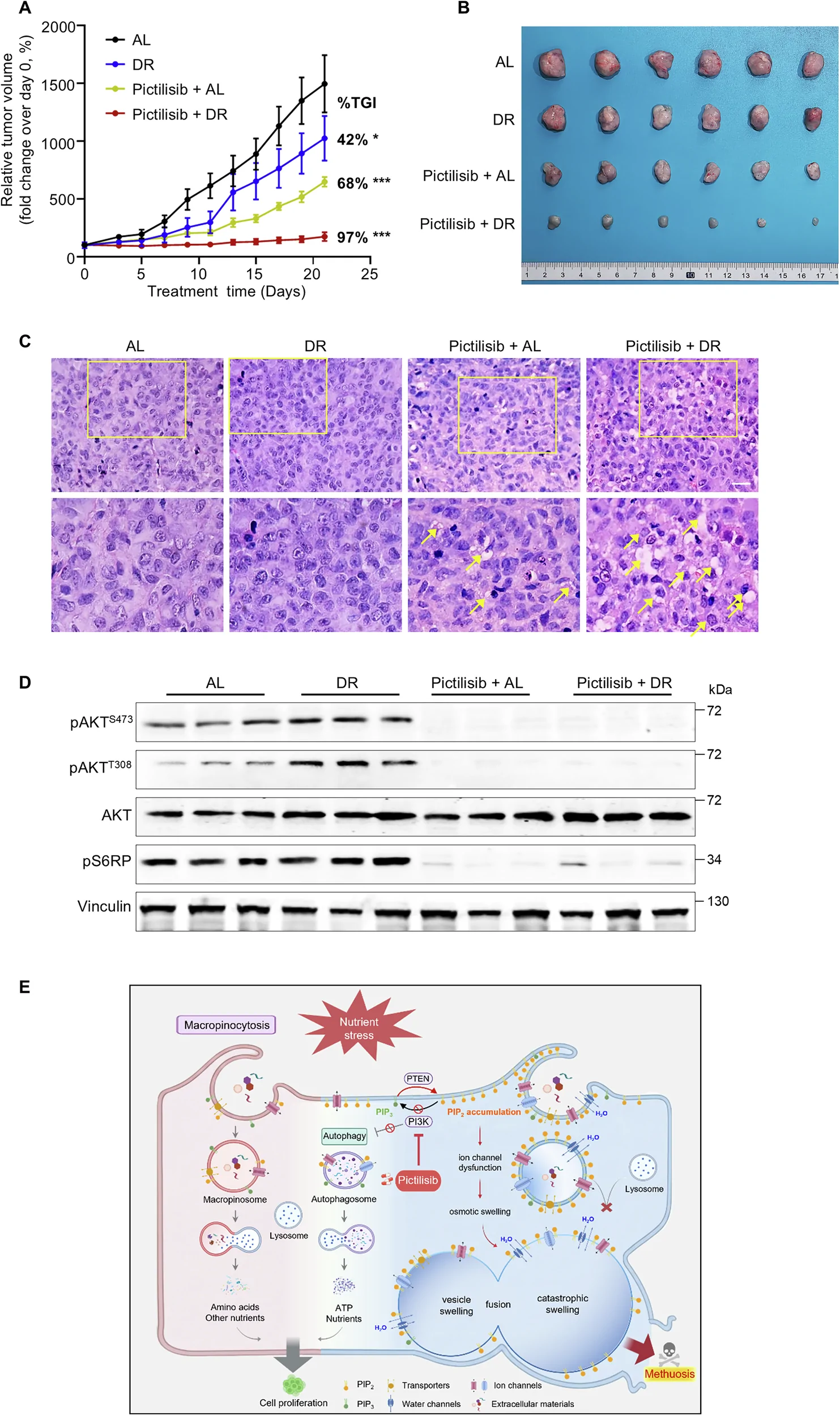
Pictilisib synergizes with dietary restriction to induce methuosis in vivo. **A** SNU119 xenograft-bearing mice were assigned to four groups for 21 days: ad libitum (AL), dietary restriction (DR), Pictilisib under AL (Pictilisib + AL), and Pictilisib with DR (Pictilisib + DR). Tumor volume fold change relative to Day 0 is shown. AL, *n* = 8; DR, *n* = 8; Pictilisib + AL, *n* = 9; Pictilisib + DR, *n* = 7. Data are shown as Mean ± SEM. *p < 0.05, ***p < 0.001 (One-way ANOVA with Tukey’s multiple comparisons test). **B** Representative endpoint tumor images for the indicated treatments. **C** Representative H&E staining of SNU119 xenograft tumors as indicated. Scale bar, 20 µm. **D** Western blot analysis of indicated proteins in tumor lysates from treated mice. Vinculin was used as a loading control. **E** Under nutrient restriction, cells activate macropinocytosis and autophagy to sustain nutrient recycling. The PI3K-PTEN axis maintains a balanced PIP_2_/PIP_3_ cycle, supports proper ion channel function and enables macropinosome and autophagosome maturation through lysosomal fusion, generating ATP and nutrients for cell proliferation and survival. Under nutrient restriction combined with Pictilisib treatment, a subset of cancer cells harboring aberrant PI3K activation maintains the capacity for macropinocytic uptake despite PI3K inhibition. Critically, macropinocytosis persists uninhibited, continuously internalizing PIP_2_-enriched plasma membrane. Without functional PIP_2_ recycling, PIP_2_ accumulates aberrantly on macropinosomal membranes. Excessive PIP_2_ dysregulates ion channels, disrupting osmolyte extrusion and generating osmotic imbalance. Water influx through aquaporins (*e.g*. AQP1) drives progressive macropinosomal swelling, resulting in catastrophic vesicle enlargement and methuotic cell death. Notably, although Pictilisib concurrently activates autophagy as a compensatory nutrient-recycling pathway, this response fails to prevent methuosis-mediated cell death under nutrient stress. These findings establish a mechanistic rationale for combining PI3K inhibition with nutrient limitation as a selective therapeutic strategy to induce methuosis in macropinocytosis-dependent tumors. Figure created with BioRender (biorender.com).

To assess the in vivo efficacy of the PI3K inhibitor Pictilisib in combination with nutrient limitation, mimicked by dietary restriction, in inducing methuosis, we established a mouse xenograft tumor model. SNU119 cells were subcutaneously inoculated into mice maintained on a standard rodent diet until tumors reached 70–80 mm^3^. Mice were then randomized into four groups: ad libitum feeding (AL), dietary-restricted (DR), Pictilisib under ad libitum conditions (Pictilisib + AL), and Pictilisib combined with dietary restriction (Pictilisib + DR). Tumor growth analysis showed that dietary restriction alone modestly suppressed tumor progression, with a tumor growth inhibition (TGI) rate of 42% (Fig. 7A, B). In contrast, Pictilisib under AL conditions significantly inhibited tumor growth (TGI of 68%; *p* < 0.001). The combination (Pictilisib + DR) elicited the strongest response, achieving a TGI of 97% (*p* < 0.001) and indicating synergy between PI3K inhibition and nutrient limitation. Histological examination of hematoxylin and eosin (H&E)-stained tumor sections revealed vacuolization in the Pictilisib + AL group, with the most extensive vacuoles in the Pictilisib + DR group (Fig. 7C, yellow arrows), consistent with methuosis induction. Immunoblotting analysis of tumor lysates confirmed effective PI3K/AKT pathway inhibition in both Pictilisib-treated groups, evidenced by marked reductions in phosphorylated AKT (Ser473 and Thr308) and phosphorylated S6RP (Ser235/Ser236) (Fig. 7D). These molecular changes correlated with the observed antitumor effects. Collectively, these in vivo findings corroborate our in vitro results, demonstrating that Pictilisib, especially when combined with dietary restriction, potently induces methuosis and suppresses tumor growth. This synergy highlights a promising therapeutic approach for cancers by exploiting methuosis through PI3K inhibition and nutrient stress.

## Discussion

PI3K inhibitors demonstrate clinical efficacy in PI3K-driven tumors but are fundamentally limited by cytostatic effects that fail to eliminate malignant cells [6–9]. Our study reveals that Pictilisib induces methuosis, a non-apoptotic and extensively vacuolized form of cell death, specifically under conditions of nutrient restriction and independently of canonical AKT signaling. These findings establish a mechanistic basis for overcoming the cytostatic limitation of PI3K inhibition through nutrient-dependent therapeutic strategies.

To identify which tumors might respond to this therapeutic approach, we stratified cell lines based on their PI3K-dependence for macropinocytosis. Cell lines capable of sustaining PI3K-independent macropinocytic flux (CaSki, ME180, MDA-MB-468, OVSAHO, SNU119) readily underwent methuotic cell death when deprived of serum. In contrast, those dependent on PI3K for macropinocytosis (PC-3, HCC1954) remained resistant. Similarly, HCC70 cells, which inherently lack macropinocytic capacity irrespective of PI3K status, showed no methuotic response. This stratification yields a critical insight that methuotic sensitivity correlates not with macropinocytic capacity per se, but specifically with the ability to maintain macropinocytosis despite PI3K inhibition under nutrient-restricted conditions.

These findings demonstrate that Pictilisib induces methuosis by blocking PI3K-catalyzed conversion of PI(4,5)P_2_ to PI(3,4,5)P_3_, resulting in PI(4,5)P_2_ accumulation at the plasma membrane. Macropinocytosis constitutively internalizes large membrane domains as vesicles, passively incorporating their lipid composition [16–18]. Since macropinocytic flux persists despite PI3K inhibition, continuous internalization of PI(4,5)P_2_-enriched plasma membrane drives progressive accumulation of this lipid on vacuolar membranes. Elevated vacuolar PI(4,5)P_2_ disrupts ion channel gating and impairs macropinosome maturation, precipitating methuotic cell death. Pharmacological hydrolysis of PI(4,5)P_2_ via PLC activation substantially attenuated vacuolization, establishing that aberrant PI(4,5)P_2_ accumulation is sufficient to initiate the methuotic program. Thus, methuosis in nutrient-restricted cells requires PI3K inhibition-driven PI(4,5)P_2_ accumulation coupled with sustained macropinocytic internalization of PI(4,5)P_2_-rich plasma membrane.

We systematically validated each step of this mechanistic cascade through convergent perturbation experiments. Pharmacological PLC activation suppressed vacuolization, confirming that elevated PI(4,5)P_2_ is sufficient to drive pathology. Inhibition of distinct ion channels with NPPB, ML133, and SET2 each prevented vacuole expansion, demonstrating that disrupted ion flux is both necessary and sufficient to suppress methuosis. Furthermore, AQP1 knockdown specifically blocked water accumulation, elucidating the osmotic consequence whereby aberrant ion influx creates osmotic pressure within macropinosomes, which AQP1 then rapidly amplifies through water uptake, progressively enlarging vacuoles until methuotic cell death ensues (Fig. 7E). To establish mechanistic specificity, we systematically evaluated whether Rac1, ROS-mediated signaling, or MKK4 activation contributed to this response [15, 43–45]. While Pictilisib did elevate ROS and MKK4 phosphorylation, neither antioxidant treatment nor MKK4 knockdown prevented vacuolization. These convergent results firmly establish that Pictilisib-induced methuosis operates through a mechanistically distinct pathway, specifically orchestrated by aberrant PI(4,5)P_2_ accumulation on macropinosomal membranes.

Morphological and functional analysis revealed that methuotic vacuoles are single-membrane macropinosomes expressing early and late endosomal markers yet failing to undergo lysosomal fusion. EIPA-mediated macropinocytosis inhibition and Bafilomycin A1-mediated vacuolar H⁺-ATPase inhibition both prevented vacuole formation in nutrient-stressed, PI3K-inhibited cells, establishing sustained macropinocytic flux as a prerequisite for methuotic progression. Although macropinocytosis has been traditionally linked to the Rac1/CDC42-PAK signaling axis [16, 42], our perturbation studies reveal that PAK is the critical effector, with PAK inhibition abrogating macropinocytic uptake while Rac1/CDC42 inhibition does not impair macropinocytic flux. This indicates that under PI3K inhibition, PAK can be activated through a pathway independent of canonical Rac1/CDC42 signaling. These findings establish PAK as the primary driver of sustained macropinocytosis under these conditions, revealing that this alternative activation mechanism permits sustained macropinocytic flux despite PI3K inhibition.

Critically, vacuolization proved reversible upon acute drug withdrawal, indicating that initial PI3K inhibition establishes a reversible cellular state. However, sustained PI3K inhibition drives an irreversible methuotic commitment, thereby defining a critical temporal threshold for therapeutic intervention. Notably, Pictilisib concurrently activates autophagy; while autophagy alone is insufficient to prevent methuosis, its inhibition sensitizes cells to methuotic death, suggesting that dual targeting of macropinocytosis and autophagy may enhance therapeutic efficacy.

To advance these mechanistic findings toward clinical relevance, in vivo studies provided crucial validation. Dietary restriction, when combined with Pictilisib, demonstrated synergistic tumor growth inhibition, confirming that both nutrient restriction and PI3K inhibition are mechanistically required to induce extensive methuosis. These results strongly establish nutrient availability as a critical contextual determinant of therapeutic response, revealing that PI3K inhibition and nutrient restriction are mechanistically coupled in driving methuotic cell death.

While this study employed pan-Class I PI3K inhibition, preliminary observations suggesting differential effects from PI3Kβ warrant rigorous isoform-specific investigation. Our findings stem from in vitro models and immunodeficient xenograft systems, which inherently lack intricate immune-tumor interactions and physiological nutrient gradients, thus limiting direct generalizability to complex tumor microenvironments. Future investigations should validate these mechanisms in patient-derived xenografts and organoid models, and biomarker-driven clinical trials incorporating assessments of macropinocytic activity and PI(4,5)P_2_ localization would be valuable. Concurrent efforts are needed to precisely define the contributions of specific PI3K isoforms to methuotic sensitivity and explore therapeutic combinations targeting macropinocytosis, ion channels, and autophagy to optimize methuotic cell death induction in PI3K-driven cancers.

## Supplementary information

Supplementary figures and figure legends

Supplemtary material_uncropped western blots

Supplemental Data 1 and 2

## Data Availability

All data generated or analyzed during this study are included in this published article and its supplementary information file. The RNA-seq data sets generated in this study have been deposited in the NCBI Gene Expression Omnibus (GEO) under accession number GSE285435.
