# Supplementary material for: Pictilisib and nutrient stress synergize to induce methuosis via PI(4,5)P2-dependent macropinocytic dysregulation in cancer cells
Source: Cell Death Dis. 2026 Jun 16;17(1):752. doi: 10.1038/s41419-026-08994-6 (PMC13500832; doi:10.1038/s41419-026-08994-6)

Figure 2A

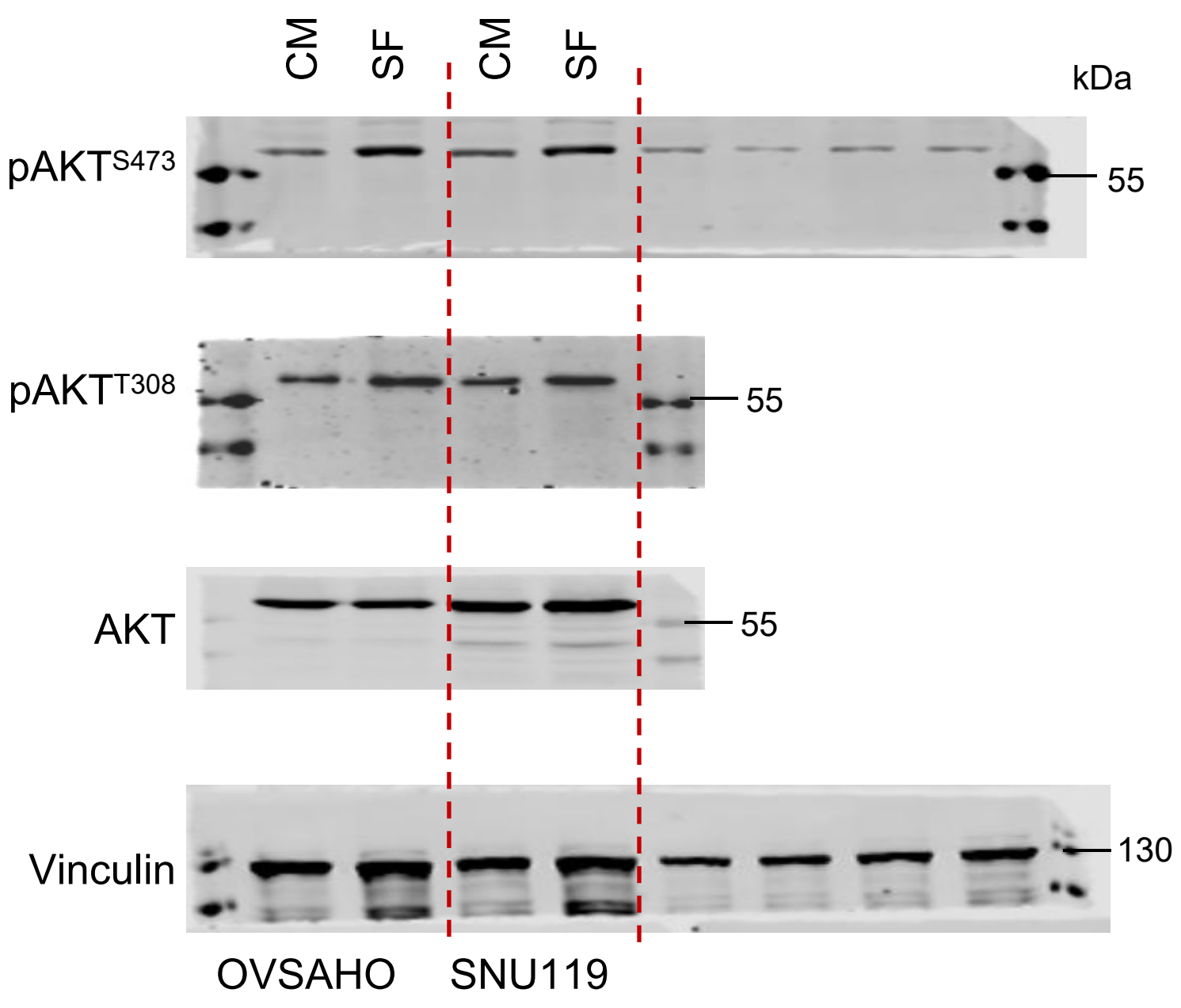

Figure 2B

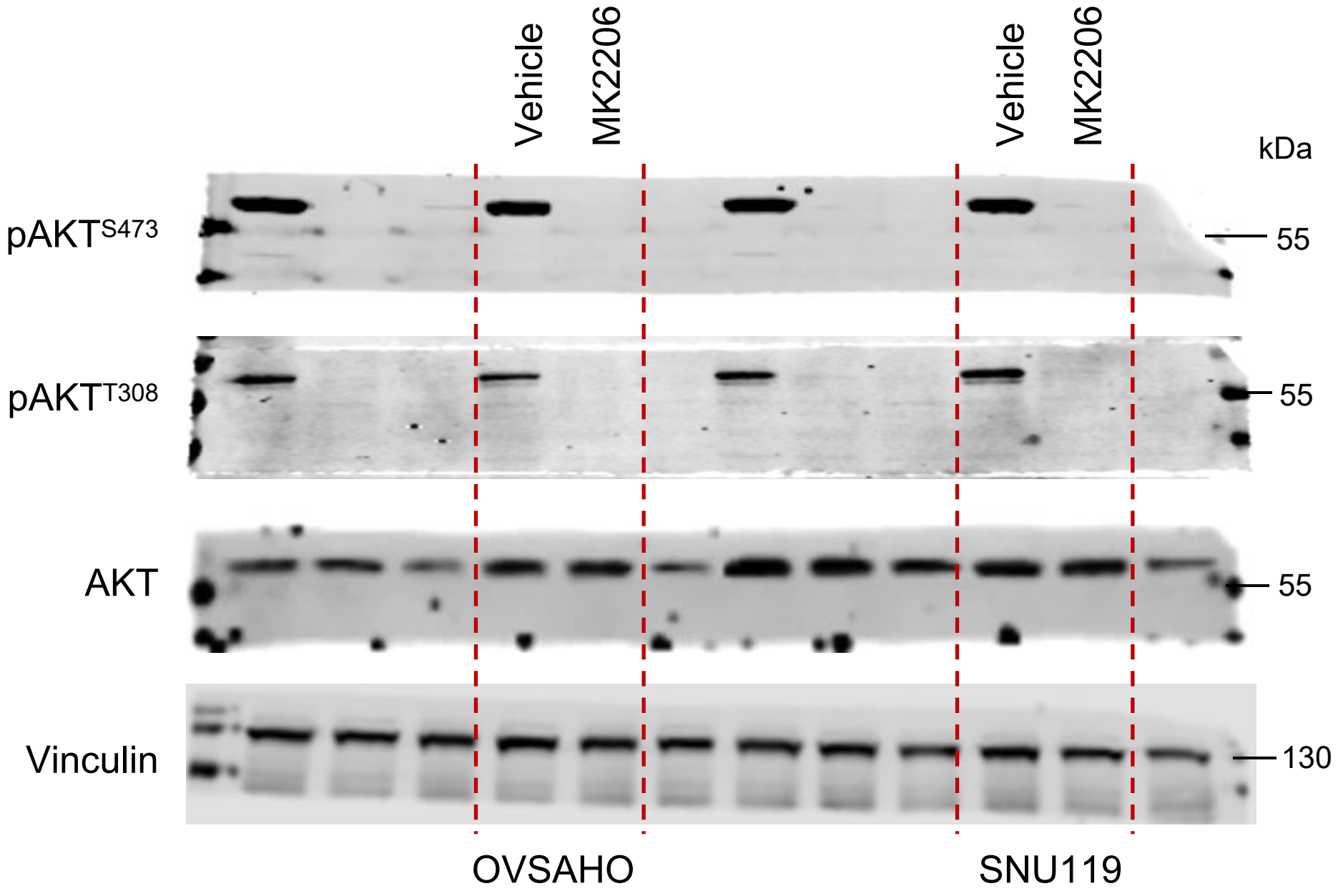

Figure 2E

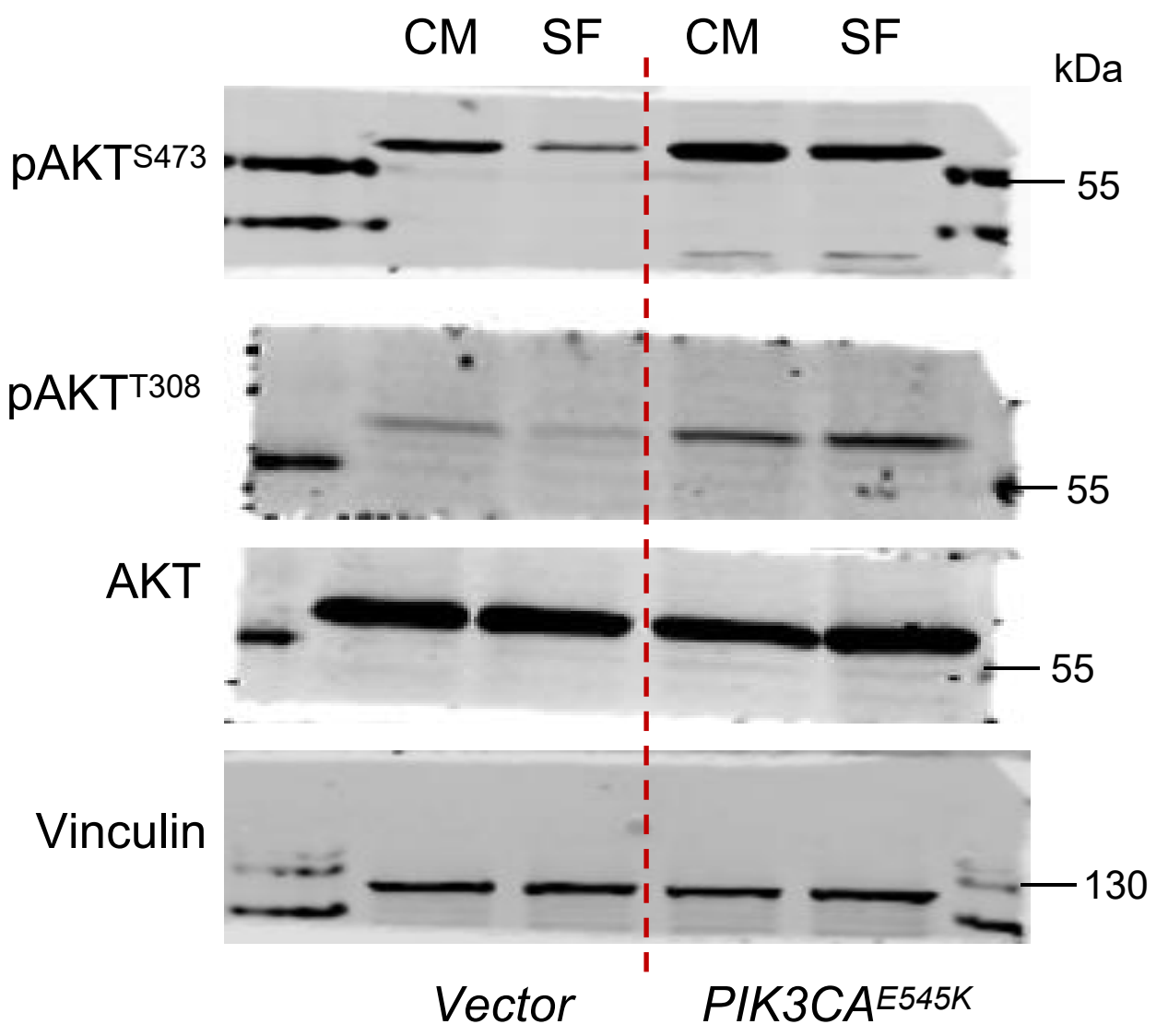

Figure 2F

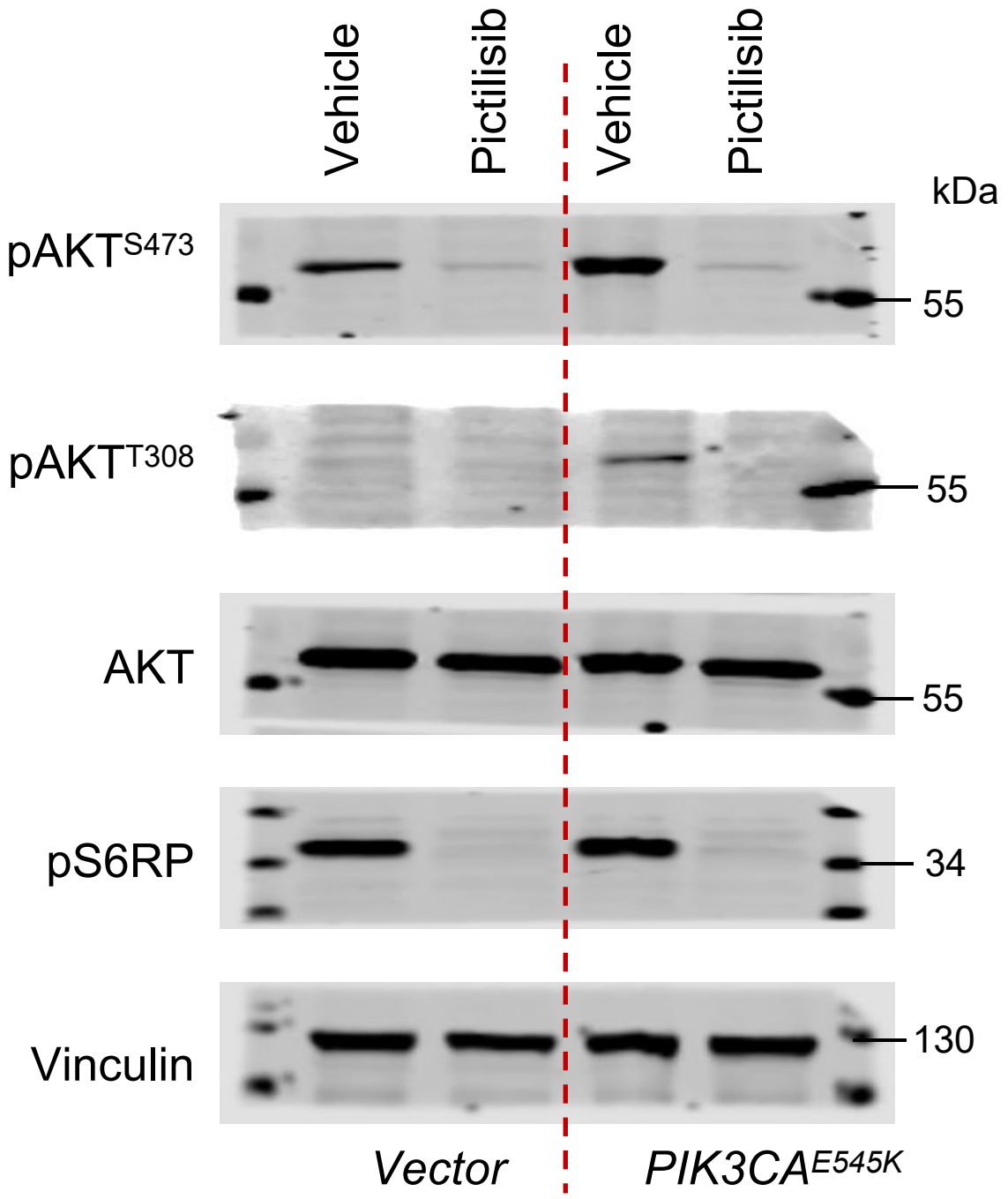

Figure 4D

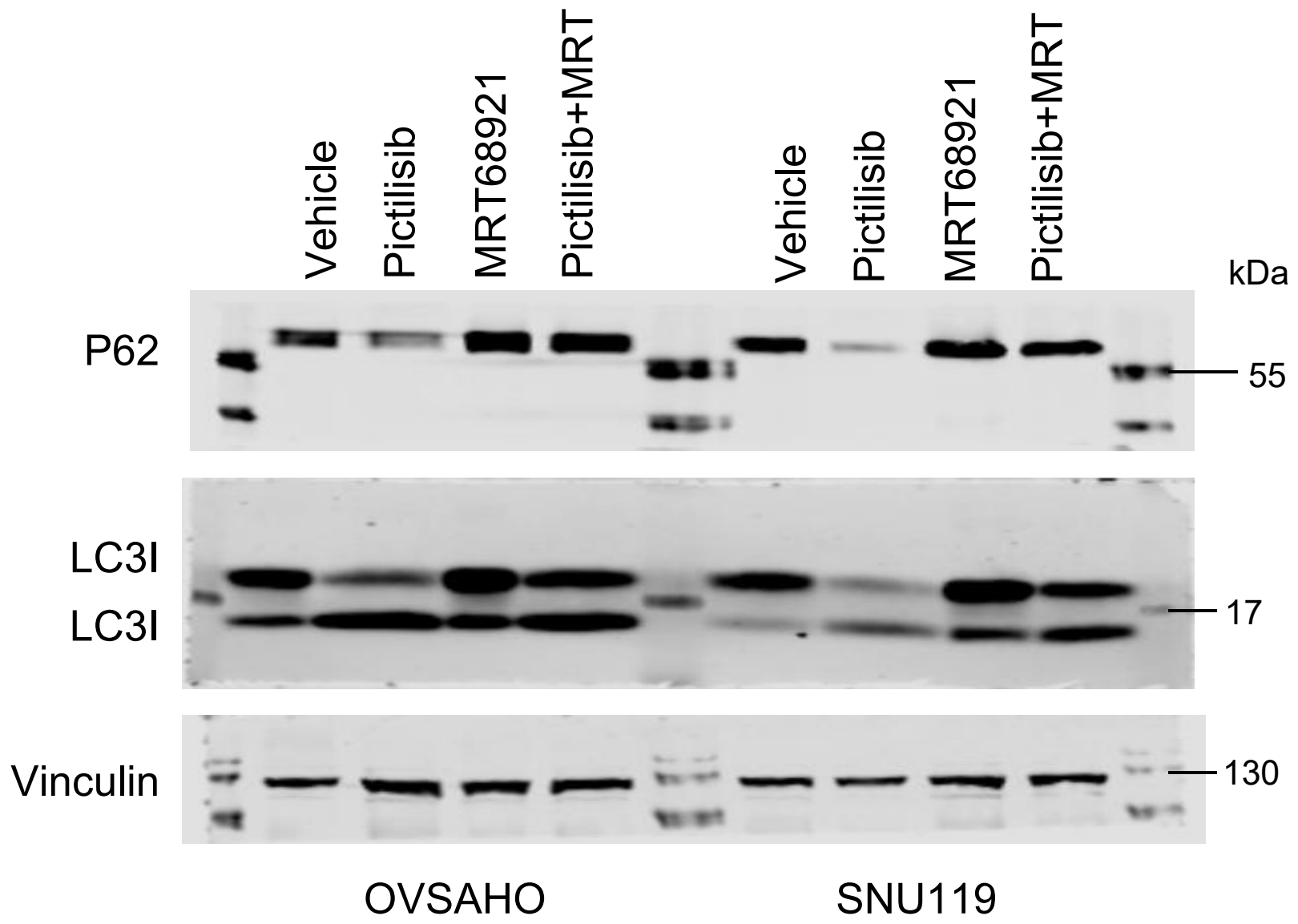

Figure 6C

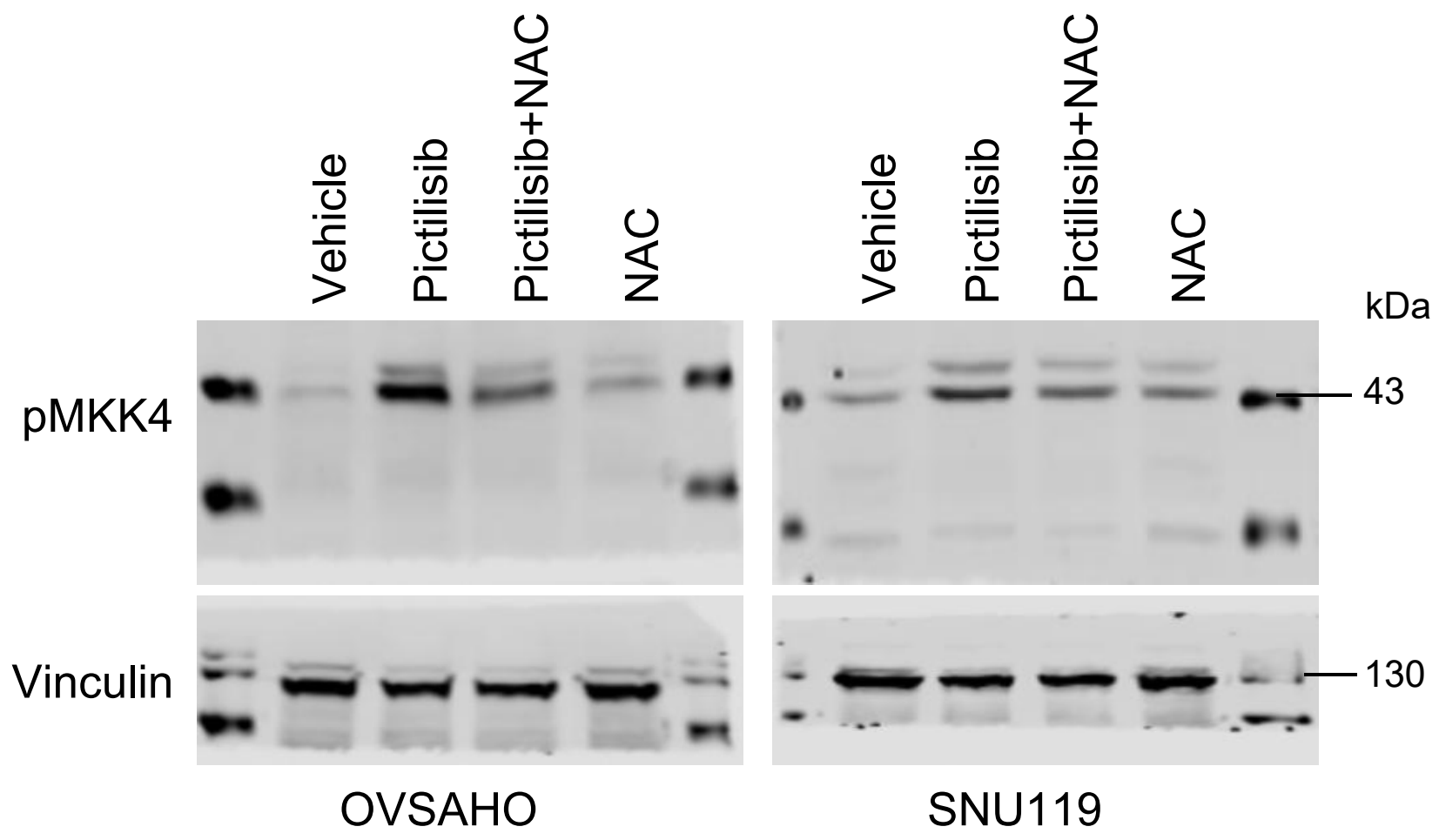

Figure 7D

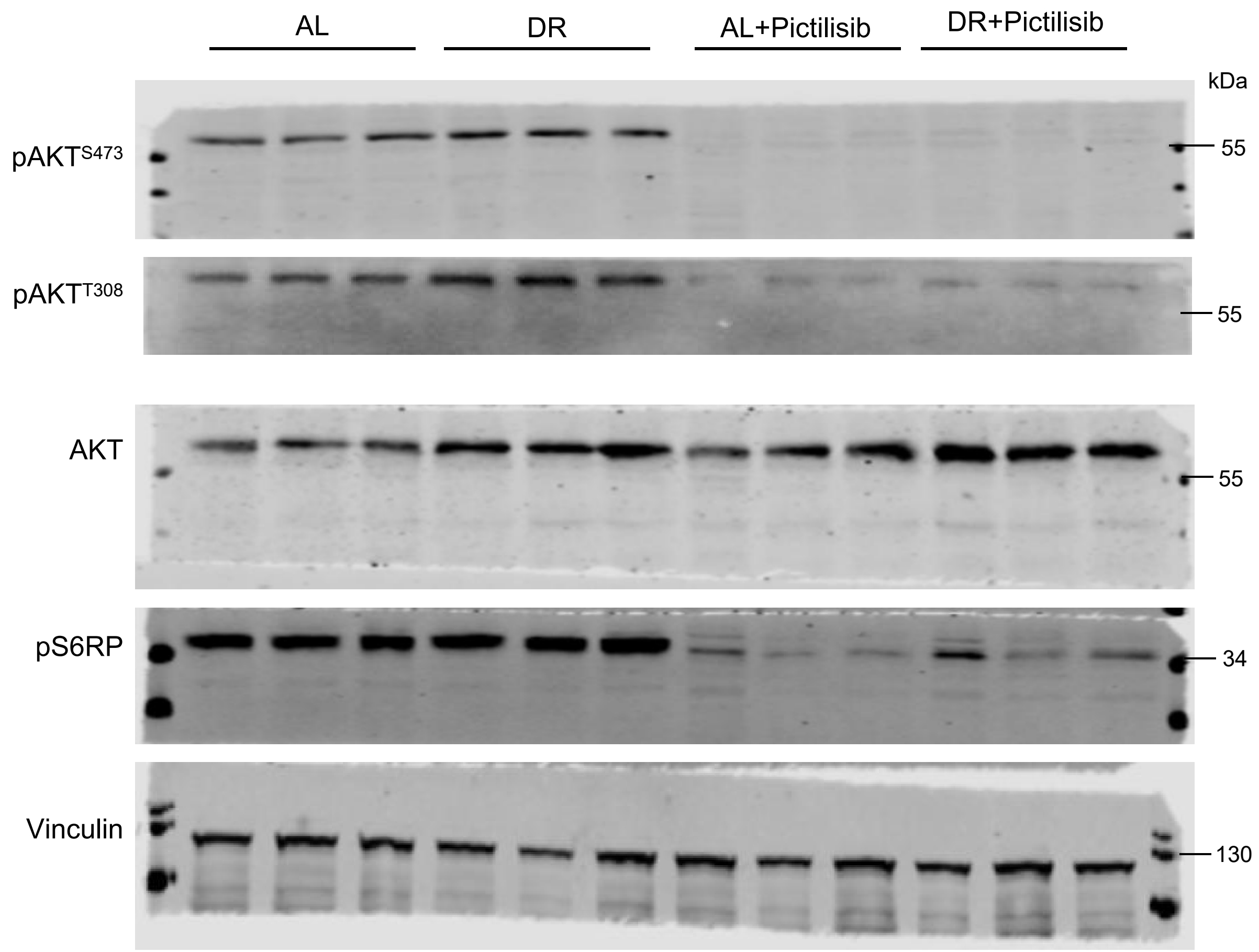
AL
DR
AL+Pictilisib
DR+Pictilisib
kDa
pAKT^{S473}
55
pAKT^{T308}
55
AKT
55
pS6RP
34
Vinculin
130

Figure S2A

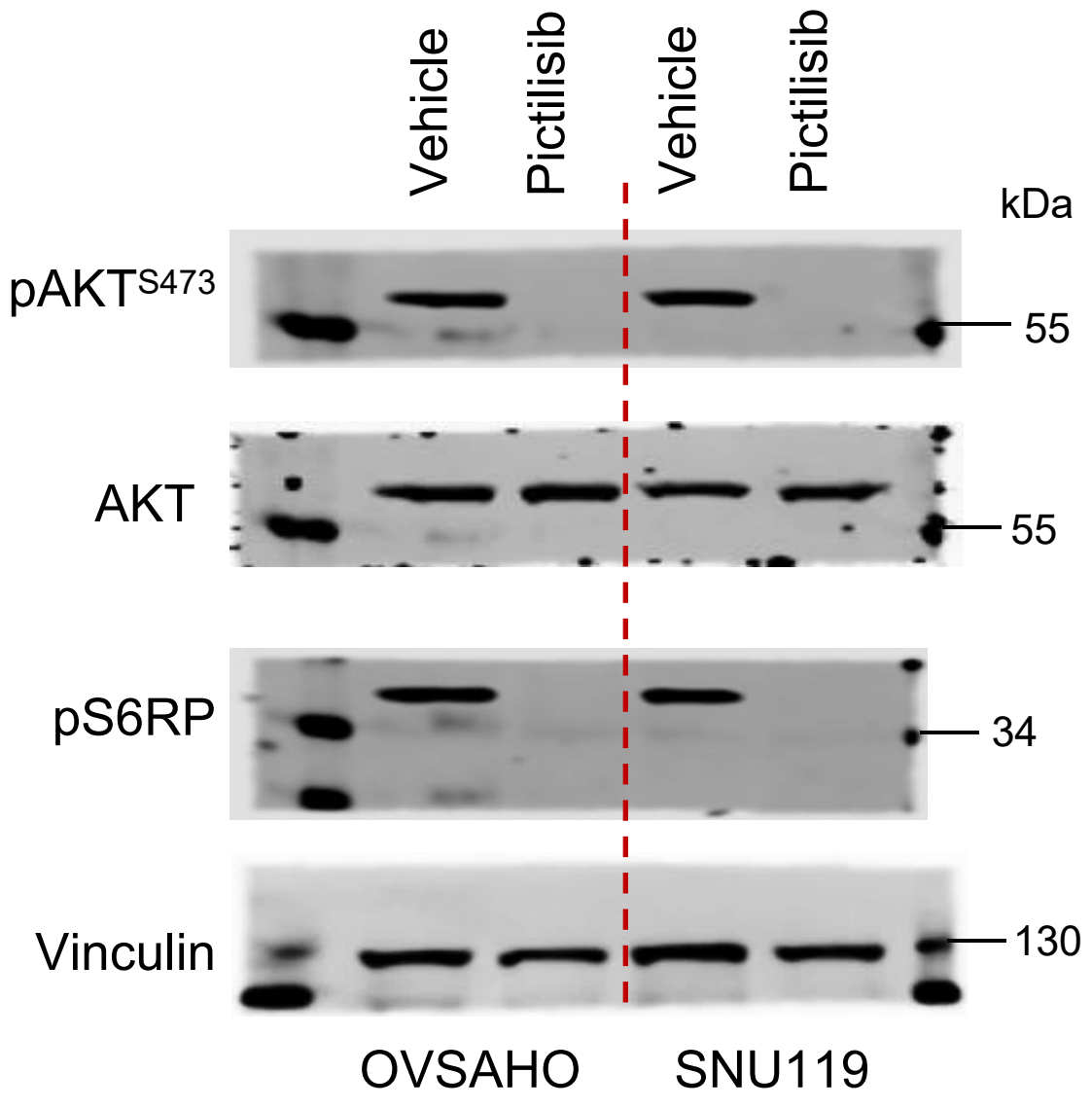

Figure S2B

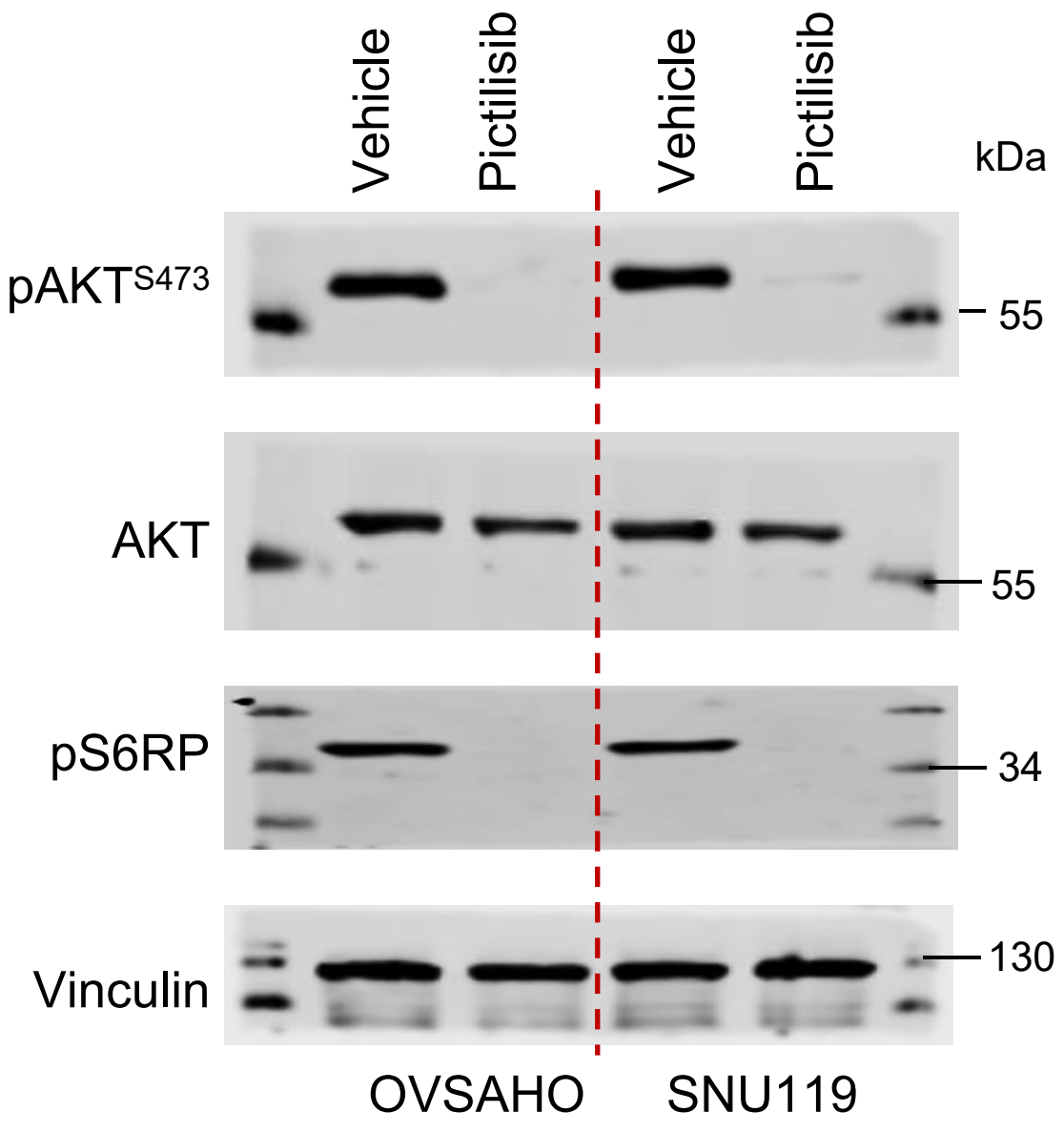

Figure S3A and B

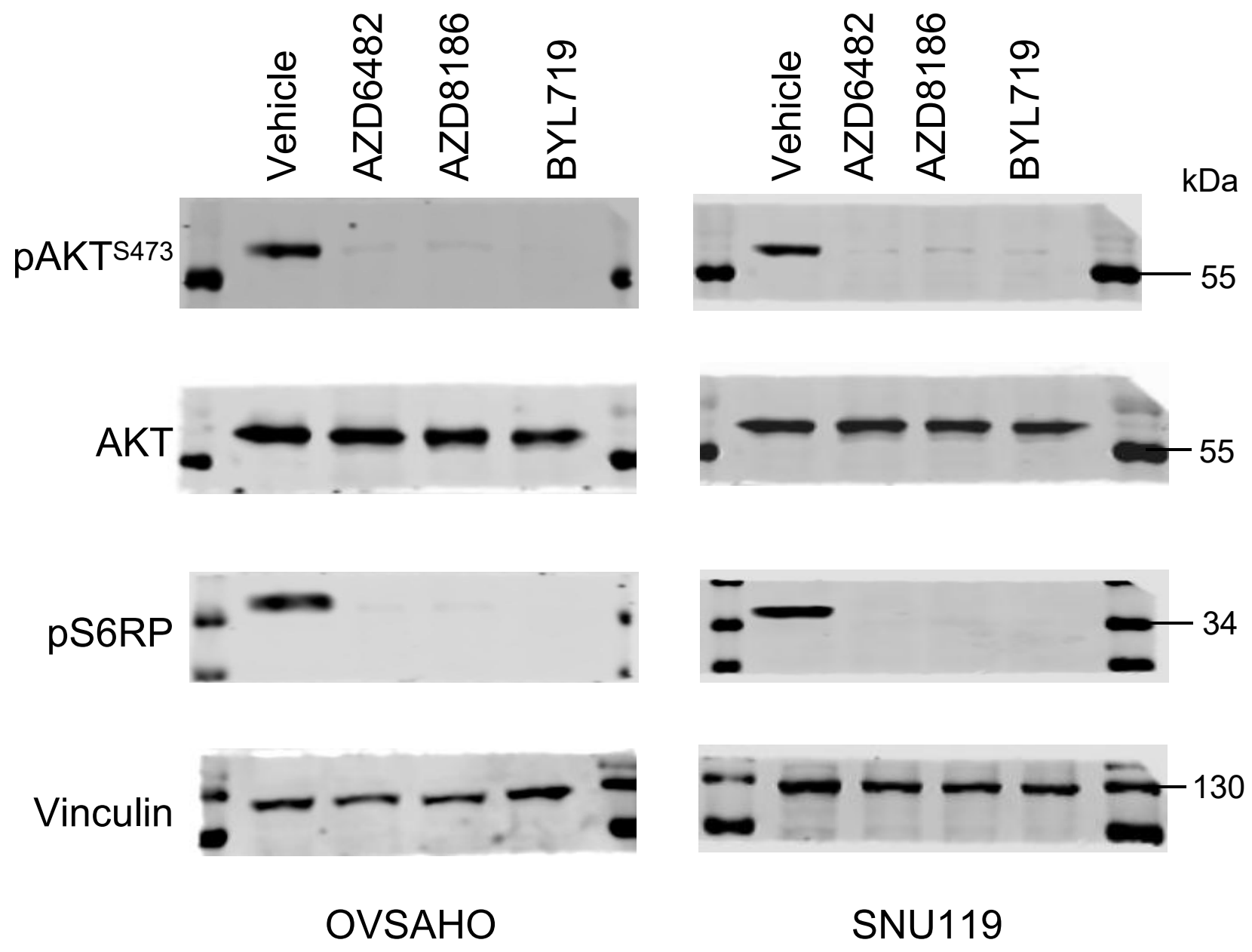

Figure S3C

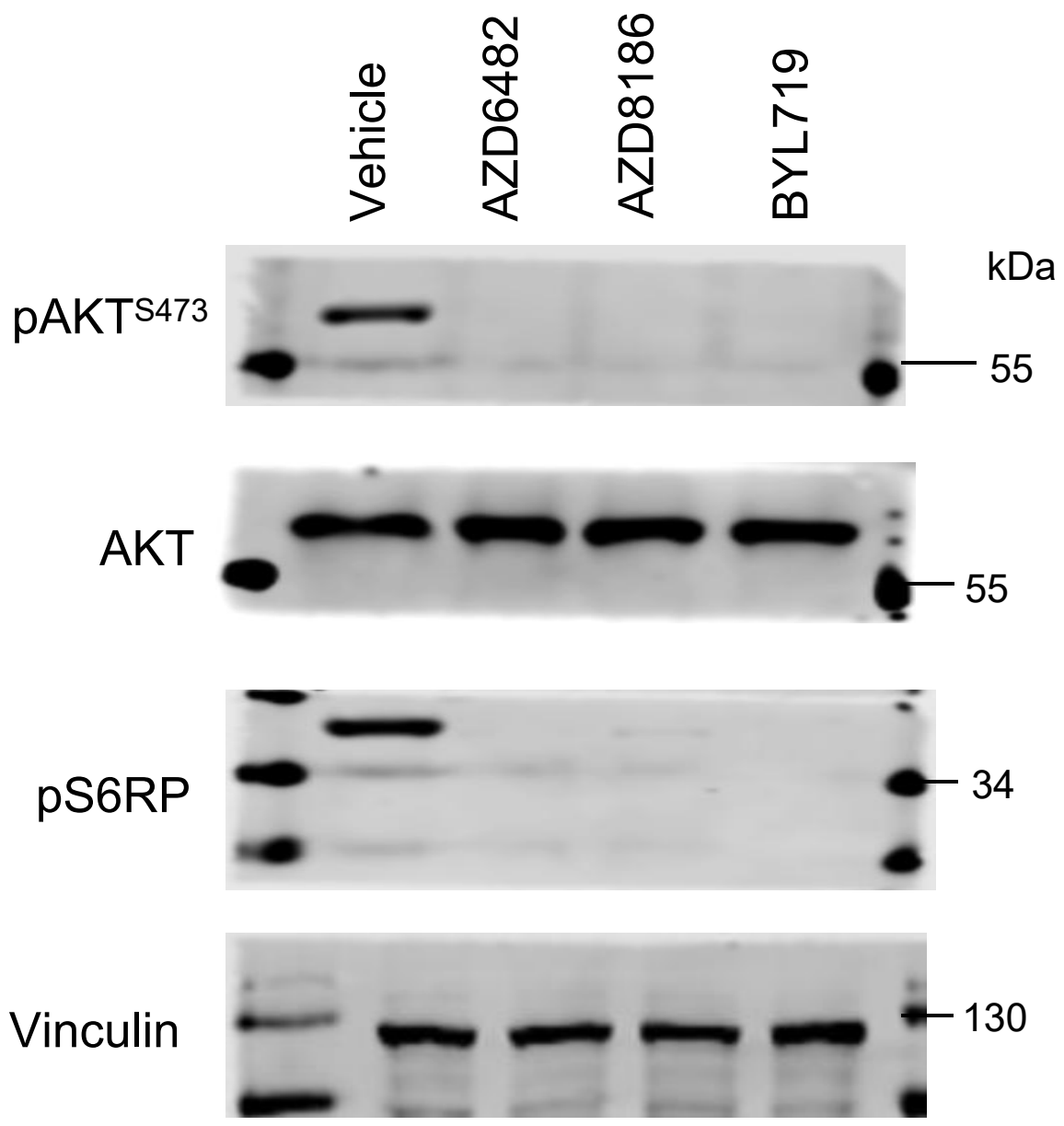

Figure S4A

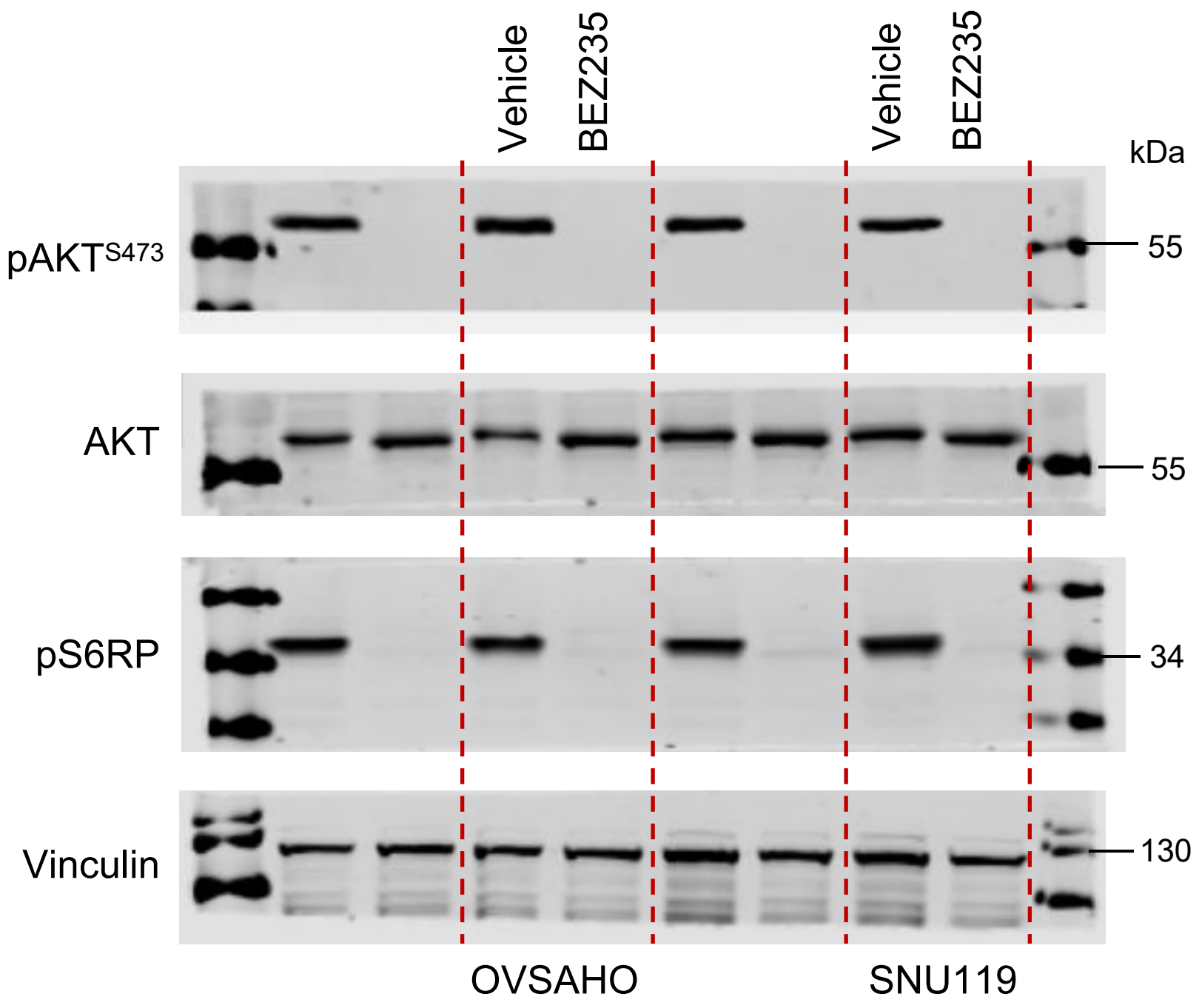

Figure S6A

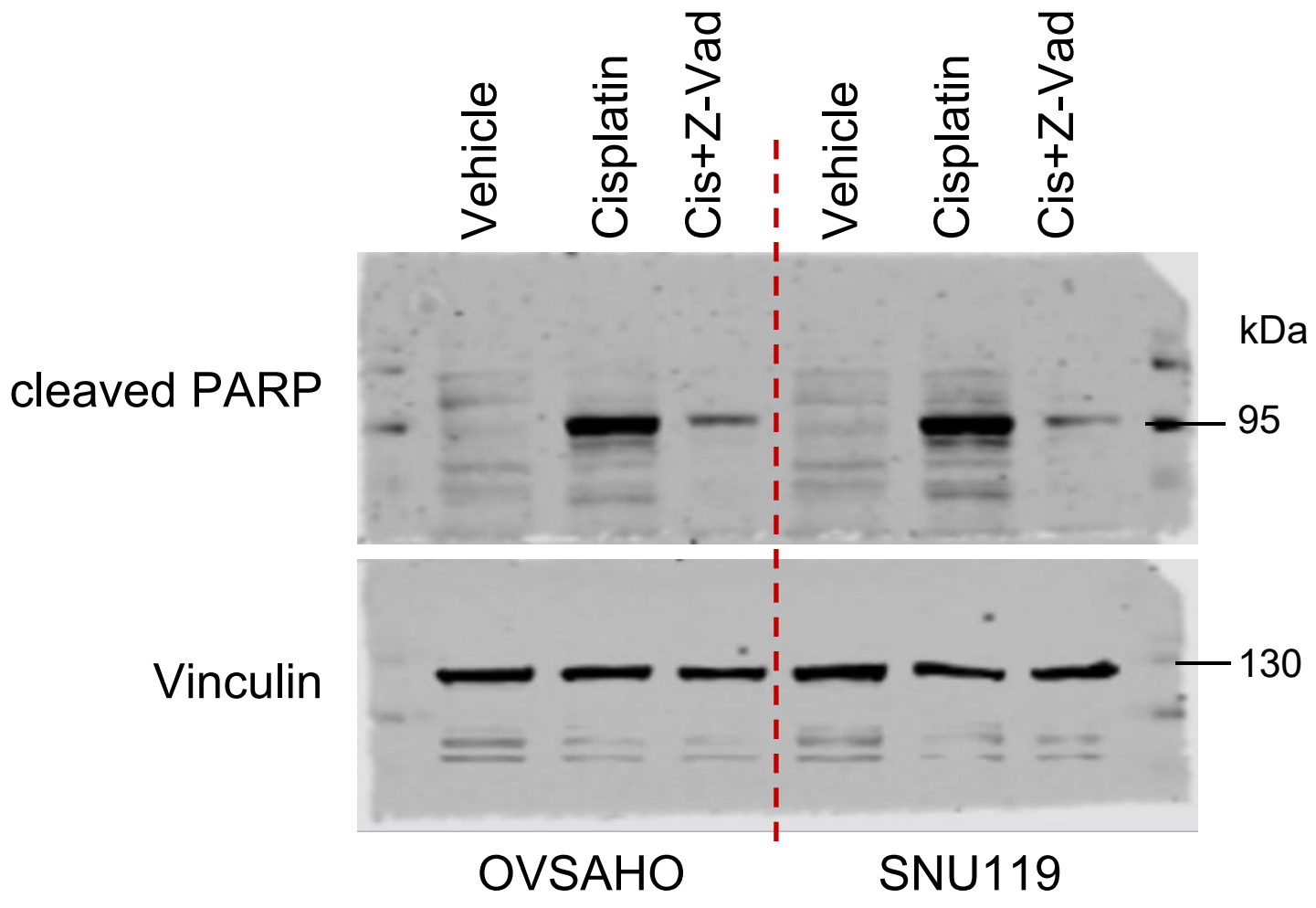

Figure S9E

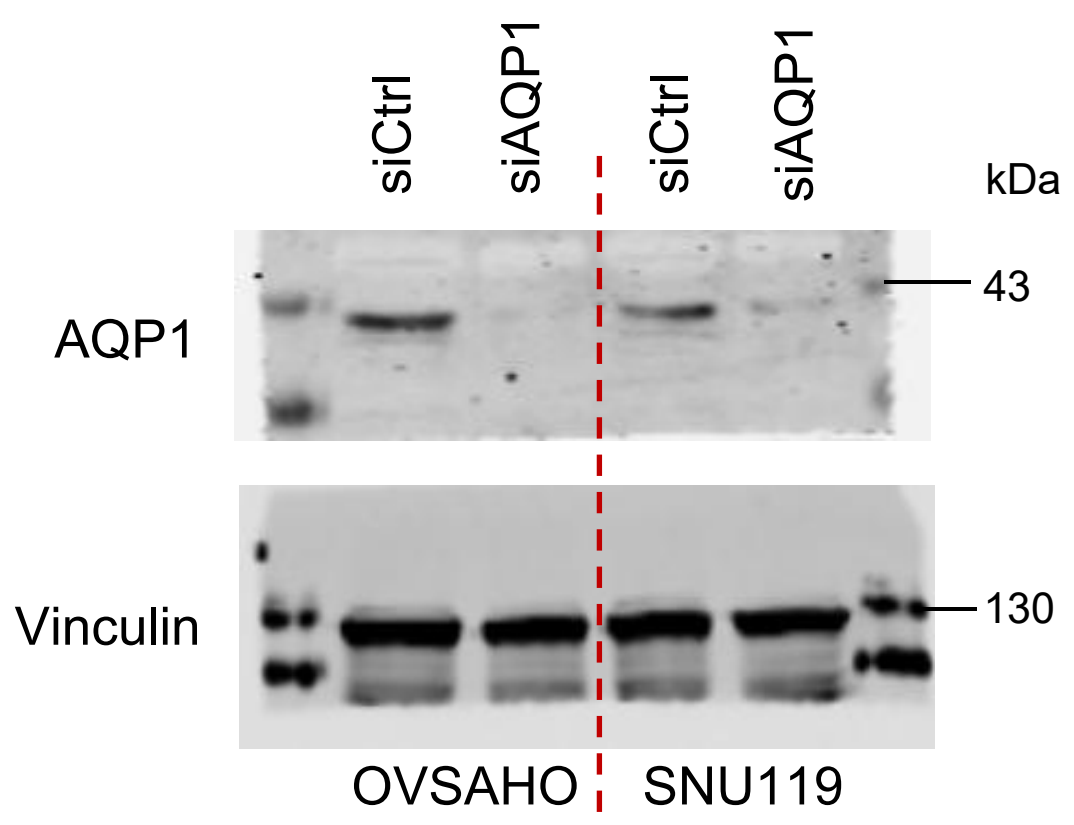

Figure S10B

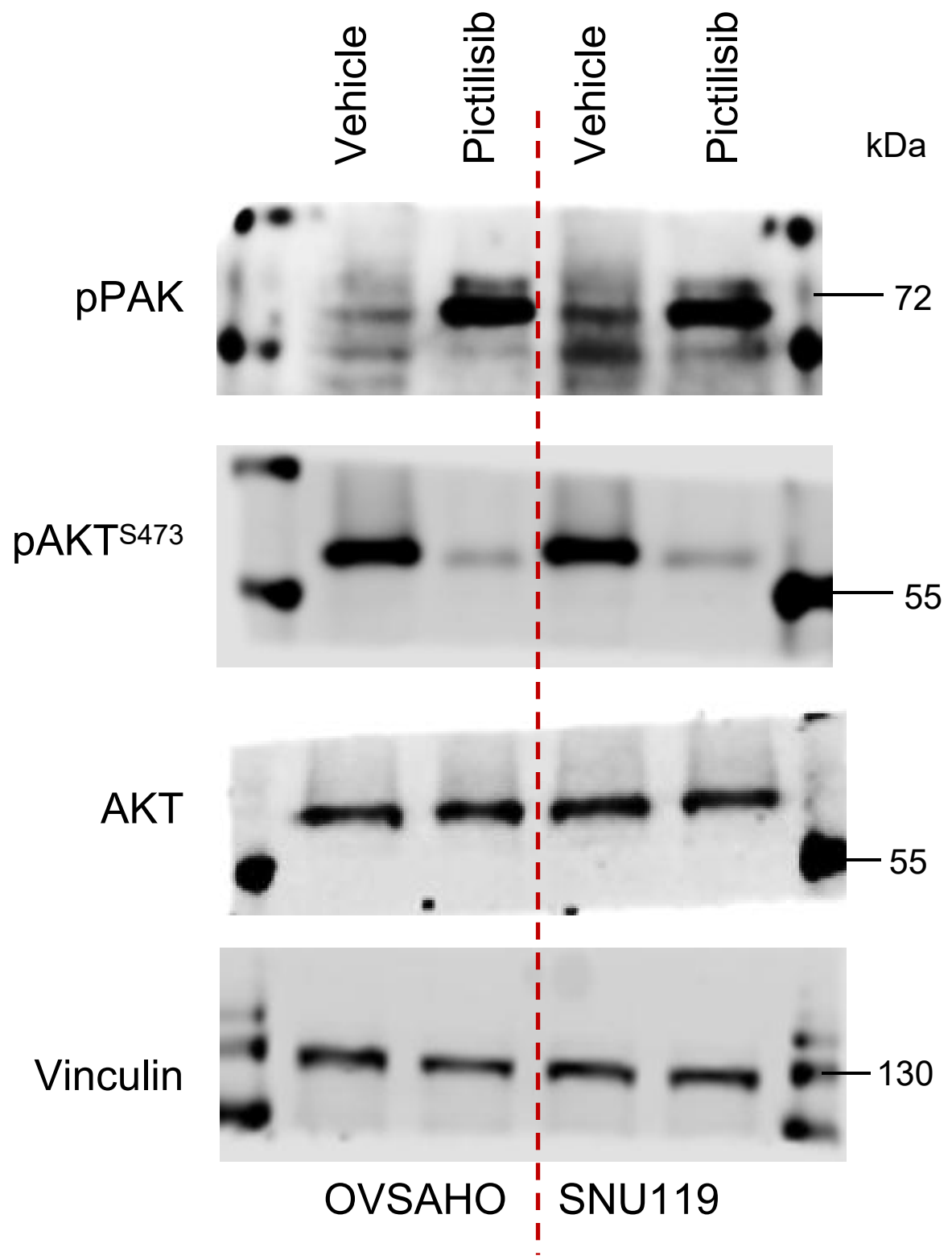

Supplement: Supplementary file 1 [file 41419_2026_8994_MOESM2_ESM.pdf]
