# Supplementary material for: Pictilisib and nutrient stress synergize to induce methuosis via PI(4,5)P2-dependent macropinocytic dysregulation in cancer cells
Source: Cell Death Dis. 2026 Jun 16;17(1):752. doi: 10.1038/s41419-026-08994-6 (PMC13500832; doi:10.1038/s41419-026-08994-6)
Supplement: Supplementary file 2 [file 41419_2026_8994_MOESM1_ESM.docx]

**Supplemental Figures and Legends**

**Fig. S1. Effects of Pictilisib on multiple cancer cell lines with alterations in the PI3K pathway. A.** TMR-Dextran uptake in multiple cancer cell lines with or without Pictilisib for 24 hours (n = 8). Scale bar, 10 µm. **B.** Phase-contrast images of cancer cells treated with Pictilisib for 24 hours (n = 4). Scale bar, 10 µm. **C.** Cell death in cancer cells treated with or without Pictilisib for 48 hours (n = 3). **D.** Clonogenic survival of cancer cells treated with or without Pictilisib for 7 days (n = 3). **C, D.** Data are shown as mean ± SD and representative of three independent experiments. ** *p* < 0.01, *** *p* < 0.001 (Student’s *t*-test). **A-D.** All experiments were conducted under serum-free conditions. Pictilisib, 5 μM.

**Fig. S2. Pictilisib induces more pronounced cytoplasmic vacuolization under serum‑free than complete medium conditions.** **A, B.** Western blot analysis of indicated proteins in OVSAHO and SNU119 cells treated with Pictilisib for 24 hours in complete medium (CM, A) and serum-free medium (SF, B). Vinculin was used as a loading control. **C.** Phase-contrast images of cytoplasmic vacuolization in OVSAHO and SNU119 cells treated with Pictilisib for 24 hours in complete medium or serum-free medium. Scale bar, 10 µm. Pictilisib, 5 μM.

**Fig. S3. Inhibition of PI3Kβ is the key trigger of cytoplasmic vacuolization in MDA-MB-468 cells. A-C.** Western blot analysis of indicated proteins in OVSAHO (A), SNU119 (B), MDA-MB-468 (C) cells treated with AZD6482 (50 µM), AZD8186 (50 µM), and BYL719 (50 µM) for 24 hours in serum-free medium. Vinculin was used as a loading control. **D.** Phase-contrast images of MDA-MB-468 cells treated as in (C). Scale bar, 10 µm. Quantification of vacuolized cells is shown as mean ± SD (n = 4) and is representative of three independent experiments. *** *p* < 0.001 (Student’s *t*-test).

**Fig. S4. The dual PI3K/mTOR inhibitor BEZ235 induces cytoplasmic vacuolization. A.** Western blot analysis of indicated proteins in OVSAHO and SNU119 cells treated with or without BEZ235 (5 µM) for 24 hours under serum-free conditions. Vinculin was used as a loading control. **B.** Phase-contrast images of cancer cells treated as in (A). Scale bar, 10 µm. Data are shown as mean ± SD (n = 4) and are representative of three independent experiments. *** *p* < 0.001 (One-way ANOVA with Tukey’s multiple comparisons test).

**Fig. S5. Bafilomycin A1 and EIPA suppress Pictilisib-induced vacuolization. A,** **B.** Phase-contrast images of OVSAHO and SNU119 cells pretreated with Bafilomycin A1 (2 nM, A) or EIPA (25 μM, B), followed by co-treatment with or without Pictilisib for 24 hours. Scale bar, 10 µm. Pictilisib, 5 μM.

**Fig. S6. Effects of Pictilisib treatment on autophagy. A.** Western blot analysis of cleaved PARP levels in cancer cells pretreated with or without Z-VAD-FMK (Z-Vad, 20 μM), followed by co-treatment with or without Cisplatin (2.5 μM) for 48 hours. Vinculin was used as a loading control. **B.** Representative fluorescence images of cancer cells stably expressing mRFP-GFP-LC3, treated with or without Pictilisib (5 μM) for 24 hours under serum-free condition. Scale bar, 10 µm.

**Fig. S7. PI(4,5)P_2_ accumulation drives Pictilisib-induced methuosis in cancer cells.** Phase-contrast and immunofluorescence images of PI(4,5)P_2_ localization in OVSAHO and SNU119 cells at indicated time points following Pictilisib treatment (5 µM). Scale bar, 20 µm. Red arrows indicate forming vacuoles.

**Fig. S8. Pictilisib induces methuosis across cancer types via aberrant PI(4,5)P_2_ enrichment on macropinosomal membranes. A.** Representative immunofluorescent images showing PI(4,5)P_2_ staining in cancer cells treated with or without Pictilisib (5 μM) for 24 hours. Scale bar, 20 µm. **B.** Phase-contrast images of cells pretreated with or without m-3M3FBS (12.5 μM), followed by Pictilisib (5 μM) treatment for 24 hours (n = 4). Scale bar, 10 µm.

**Fig. S9. Inhibition of multiple ion channels suppresses Pictilisib-induced cytoplasmic vacuolization in cancer cells.** **A**-**C.** Phase-contrast images of OVSAHO and SNU119 cells pretreated with NPPB (80 μM, A), ML133 (25 μM, B), or SET2 (10 μM, C), followed by co-treatment with or without Pictilisib for 24 hours (n = 4). Scale bar, 10 µm. **D.** Phase‑contrast images of cancer cells transfected with siCtrl or siAQP1 for 48 hours, followed by co‑treatment with or without Pictilisib for 24 hours. Scale bar, 10 µm. **E.** Western blot analysis of AQP1 in cells transfected with siCtrl or siAQP1 for 72 hours. Vinculin was used as a loading control.

**Fig. S10. PAK is the primary driver of PI3K-independent macropinocytotic uptake in cancer cells.** **A.** OVSAHO and SNU119 cells were pretreated with the RAC1 inhibitor NSC23766 (10 μM), CDC42 inhibitor ML141 (5 μM), PAK inhibitor or FRAX597 (0.5 μM), followed by co-treatment with or without Pictilisib for 24 hours. Representative fluorescence images of TMR-Dextran uptake are shown. Scale bar, 10 µm. **B.** Western blot analysis of proteins as indicated in OVSAHO and SNU119 cells treated with Pictilisib (5 μM) for 48 hours under serum-free condition. Vinculin was used as a loading control.

**Fig. S11. Pictilisib-induced methuosis is independent of the ROS-MKK4 pathway. A.** Phase-contrast images showing cytoplasmic vacuolization in cancer cells pretreated with NAC (4 mM), followed by cotreatment with Pictilisib for 24 hours (n = 4). Scale bar, 10 µm. **B.** qRT-PCR analysis of *MKK4* mRNA in cells transfected with siCtrl or siMKK4 for 48 hours. *ACTB* served as the endogenous control. Data are shown as mean ± SD (n = 3) and representative of three independent experiments. *** *p* < 0.001 (Student’s *t*-test). **C.** Phase-contrast images showing cytoplasmic vacuolization in cells transfected with siCtrl or siMKK4 for 48 hours, followed by Pictilisib treatment for 24 hours (n = 4). Scale bar, 10 µm. **A-C.** All experiments were conducted under serum-free conditions. Pictilisib, 5 μM.
